# Sex Chromosome-wide Transcriptional Suppression and Compensatory *Cis-*Regulatory Evolution Mediate Gene Expression in the *Drosophila* Male Germline

**DOI:** 10.1371/journal.pbio.1002499

**Published:** 2016-07-12

**Authors:** Emily L. Landeen, Christina A. Muirhead, Lori Wright, Colin D. Meiklejohn, Daven C. Presgraves

**Affiliations:** 1 Department of Biology, University of Rochester, Rochester, New York, United States of America; 2 The Ronin Institute, Montclair, New Jersey, United States of America; 3 School of Biological Sciences, University of Nebraska, Lincoln, Nebraska, United States of America; Adolf Butenandt Institute, GERMANY

## Abstract

The evolution of heteromorphic sex chromosomes has repeatedly resulted in the evolution of sex chromosome-specific forms of regulation, including sex chromosome dosage compensation in the soma and meiotic sex chromosome inactivation in the germline. In the male germline of *Drosophila melanogaster*, a novel but poorly understood form of sex chromosome-specific transcriptional regulation occurs that is distinct from canonical sex chromosome dosage compensation or meiotic inactivation. Previous work shows that expression of reporter genes driven by testis-specific promoters is considerably lower—approximately 3-fold or more—for transgenes inserted into X chromosome versus autosome locations. Here we characterize this transcriptional suppression of X-linked genes in the male germline and its evolutionary consequences. Using transgenes and transpositions, we show that most endogenous X-linked genes, not just testis-specific ones, are transcriptionally suppressed several-fold specifically in the *Drosophila* male germline. In wild-type testes, this sex chromosome-wide transcriptional suppression is generally undetectable, being effectively compensated by the gene-by-gene evolutionary recruitment of strong promoters on the X chromosome. We identify and experimentally validate a promoter element sequence motif that is enriched upstream of the transcription start sites of hundreds of testis-expressed genes; evolutionarily conserved across species; associated with strong gene expression levels in testes; and overrepresented on the X chromosome. These findings show that the expression of X-linked genes in the *Drosophila* testes reflects a balance between chromosome-wide epigenetic transcriptional suppression and long-term compensatory adaptation by sex-linked genes. Our results have broad implications for the evolution of gene expression in the *Drosophila* male germline and for genome evolution.

## Abstract 2

Expression of sex-linked genes in the *Drosophila* male germline reflects a balance between an X chromosome-wide transcriptional suppression and long-term, gene-wise evolutionary recruitment of strong, compensatory promoter elements.

## Author Summary

The evolution of different sex chromosomes (e.g., *X* and *Y*) has occurred many times in animals and plants. One consequence of having different chromosome copy numbers between the sexes (*XY* males and *XX* females) is the evolution of sex chromosome-specific regulation, both in the soma (i.e., X chromosome dosage compensation) and in the male germline (i.e., meiotic sex chromosome inactivation). Understanding how the X is regulated in the male germline has implications for gene expression, the evolution of sex chromosome-specific gene content, and speciation. Surprisingly, how the X chromosome is regulated in the *Drosophila melanogaster* male germline remains unclear. We have characterized X suppression, a novel form of X chromosome transcriptional regulation specific to the *Drosophila* male germline. Our results reveal that transcription of the X is suppressed 2- to 4-fold for endogenous genes. We show that the X chromosome has evolved strong testis-specific promoters via the gene-by-gene recruitment of sequence elements that counteract transcriptional suppression of the X chromosome. These findings reveal a novel form of X chromosome regulation and lead to a new model for the control of gene expression in the *Drosophila* male germline.

## Introduction

Heteromorphic sex chromosomes—e.g., *XY* males in *Drosophila* and mammals and *ZW* females in birds and butterflies—have evolved independently numerous times in animals and in plants [1,2]. The different chromosome copy numbers between the sexes and the general lack of recombination between *X* and *Y* (*Z* and *W*) chromosomes have resulted in the evolution of sex chromosome-specific gene contents, rates of mutation, rates of evolution, and chromosome-wide forms of regulation [3–8]. Two types of sex chromosome regulation have evolved independently in disparate taxa: sex chromosome dosage compensation, a process that results in roughly equal X:autosome expression levels between the sexes [9,10], and meiotic sex chromosome inactivation (MSCI), the precocious heterochromatinization and transcriptional silencing of the sex chromosomes during meiosis I in the heterogametic sex [11–13].

Sex chromosome dosage compensation has evolved in taxa with *XY* (*Drosophila*, mammal), *XO* (nematode), and, to varying degrees, *ZW* systems [14–17]. While the mode and molecular basis of dosage compensation differs among taxa, the function is the same [10,18]. In the somatic cells of *Drosophila melanogaster* males, the single X chromosome is dosage compensated by two mechanisms. First, generic basal dosage compensation mechanisms—including buffering and gene-specific regulation—result in an average ~1.5-fold increase in expression from the X [19]. Second, sex chromosome-specific dosage compensation up-regulates X-linked genes a further ~1.35-fold via the recruitment of the Male-Specific Lethal (MSL) protein-RNA complex to chromatin entry sites enriched for a GA-rich ~21-bp MSL recognition element (MRE) [20,21]. In several *Drosophila* lineages, neo-X chromosomes—i.e., ancestral autosomes that now segregate as sex chromosomes—have independently co-opted MSL-mediated dosage compensation via the de novo evolution of MREs [22–24].

MSCI has also evolved independently in taxa with *XY* (e.g., mammal) and *XO* systems (e.g., nematode, grasshoppers [25–27]); it is unclear if MSCI acts in *ZW* systems [28, 29]. During MSCI in *XY* and *XO* systems, sex chromosomes are sequestered into a subcompartment of the nucleus and decorated with epigenetic modifications characteristic of heterochromatin formation and/or transcriptional silencing [26–29]. In mice, MSCI strongly impacts gene expression, resulting in the ~10-fold down-regulation of ~80% of X-linked genes in spermatocytes [30,31]. Like sex chromosome dosage compensation, the molecular basis of MSCI differs among taxa [12,13], but, unlike dosage compensation, the function of MSCI is still unclear [13]. It has been suggested that MSCI is an epigenetic form of host genome defense against selfish genetic elements [13,32,33] or that it functions to prevent recombination events between non-homologous X and Y chromosomes [34].

How sex chromosome gene expression is regulated in the *Drosophila* male germline has proved surprisingly difficult to resolve. Despite early claims that the X chromosome and autosomes are expressed at similar levels (e.g., [35,36]), sex chromosome-specific dosage compensation appears absent in the *Drosophila* male germline. First, key components of the MSL complex are not expressed in testes, and those that are do not localize to the X chromosome, indicating a lack of MSL-mediated sex chromosome dosage compensation [37–39]. Second, median expression of the X chromosome is ~1.5-fold lower relative to autosomes, consistent with basal but not sex chromosome dosage compensation [40–42]. Similarly, MSCI may also be absent from the *Drosophila* male germline, as previous data from cytology, microarray analyses, and indirect genetic evidence have failed to settle the question. Direct cytological evidence is inconclusive or lacking [34,43–46], and microarray analyses do not demonstrate the expected strong global down-regulation of X-linked gene expression as cells progress from premeiotic to meiotic stages of spermatogenesis ([40,47]; but see [48,49]).

Two genetic findings have been suggested as evidence for MSCI in *Drosophila*. First, ~75% of X-autosome reciprocal translocations cause dominant male sterility (autosome-autosome translocations do not), as might be expected if putative allocyclic condensation of the sex chromosomes, and hence MSCI, is disrupted ([50]; but see Results below). Second, and more direct, the expression levels of transgene reporters in the *Drosophila* male germline are consistently lower for X-linked insertions than autosomal ones ([51–53]; see also [54]). In particular, promoters from five genes (two autosomal, three X-linked) with normally strong testis expression have been found to drive 3- to 8-fold lower expression of the *lacZ* reporter when the transgenes reside on the X chromosome ([51–53]; see also [54]). If the X chromosome undergoes MSCI, then X-linked transgenes may be prematurely silenced in primary spermatocytes, yielding lower average expression than autosomal transgenes [51,54]. The transgene findings are compelling, but some aspects of the data are difficult to reconcile with MSCI. For one, endogenous X-linked genes are not expressed ≥3-fold lower than autosomal genes in testes [40,41,48]. For another, RNA in situ analyses show that some X-linked transgene reporters initiate transcription relatively late in primary spermatocytes—i.e., at precisely the stage that MSCI is expected to silence the X [53]. Finally, the transcriptional suppression of some X-linked transgene reporters is detectable early in the male germline, in cells enriched for mitotic gonialblasts, prior to any putative MSCI [40]. Thus, while the transcriptional suppression of X-linked transgenes—which, for convenience, we hereafter term X suppression—is a real and robust phenomenon, it probably does not correspond to canonical MSCI (as in mammals or worms).

Here we further characterize the regulation of X chromosome gene expression in the *Drosophila* male germline. First, we test if X suppression is restricted to genes with testis-specific promoters or is more general. Second, we test if X suppression is limited to transgene constructs having transposable elements as vectors—i.e., does X suppression correspond to a form of transposon silencing that differs between the X and autosomes? Third, we test if X suppression is specific to the male germline. Fourth, we test if X-autosome translocations show evidence of X suppression (or MSCI) in *Drosophila* testes. Finally, we present evidence that X-linked genes have adapted to X suppression via the recruitment of strong testis-specific promoters. We computationally identify and then functionally validate a promoter element that drives strong expression in the testis, is especially enriched in the promoters of testis-specific genes on the X chromosome, and is evolutionarily conserved. Our results reveal that the X chromosome has evolved strong testis-specific promoters via the gene-by-gene recruitment of sequence elements that counteract sex chromosome-wide transcriptional suppression in the *Drosophila* male germline. The strong promoters on the X chromosome effectively compensate the effects of transcriptional suppression, rendering X suppression undetectable except via genetic manipulations that move genes between the X and autosomes. These findings lead to a new model for the control of gene expression in the male germline and have clear implications for the evolution of gene expression, gene duplication, and gene location in the genome.

## Results

### X Suppression Is Not Limited to Testis-Specific Promoters

All previous evidence for transcriptional suppression on the X chromosome in the *Drosophila* male germline (hereafter, “X suppression”) has come from the study of *P*-element transgenes in which testis-specific promoters drive the expression of reporter genes [51,53,54]. It is therefore unclear if X suppression is restricted to testis-specific promoters or affects all promoters. We therefore tested if promoters that drive less tissue-specific expression profiles are subject to X suppression. We first confirmed X suppression for *lacZ* transgene reporters driven by the promoter of the autosomal testis-specific gene *ocnus* with a subset of X-linked and autosomal inserts used in previous work [51]: *ocnus* transgenes inserted into X chromosome locations (*n* = 5) are expressed 14.5-fold lower than those inserted into autosomal ones (*n* = 5; Table 1). To test if X suppression occurs for less tissue-specific promoters, we assayed testis expression of mini-*white* for the same transgenes (*white* is expressed in the male germline [40]): mini-*white* is expressed 1.7-fold lower from X-linked transgenes than autosomal transgenes (Table 1). Next, to test if X suppression affects promoters that mediate broad expression profiles, we assayed testis expression of transgene reporters driven by *Actin 5c* (*Act5c*) and *Ubiquitin* (*Ubi*). As Table 1 shows, *Act5c* and *Ubi* transgenes are expressed 23.2-fold and 9.6- to 13.8-fold lower on the X compared to autosomes. These results show, for a small sample of promoters (but see below), that X suppression is not limited to genes with testis-specific expression.

**Table 1:** Testis expression of transgene reporters driven by testis-specific and non-testis–specific promoters.

Promoter	Transgene	X-linked^a^	*n*_X_^b^	SE_X_	Autosomal^a^	*n*_A_^b^	SE_A_	fold-change	*p-*Value^c^
*ocnus*	*P{wFl-ocn-lacZ}*	-6.924	5	0.329	-3.071	5	0.334	14.5	**2.5E-06**
mini-*white*	*P{wFl-ocn-lacZ}*	-8.510	5	0.219	-7.769	5	0.133	1.7	**2.9E-03**
*Actin 5c*	*P{Act-GFP}JMR*	-1.322	1	0.074	3.216	3	0.222	23.2	**2.2E-12**
*Ubiquitin*	*P{Ubi-GFP(S65T)nls}*	-10.161	2	0.445	-6.373	2	0.997	13.8	**2.9E-05**
	*P{Ubi-GFP*.*D}*	5.121	2	0.285	8.380	2	3.260	9.6	**5.1E-06**

^a^ Log2 expression relative to control gene expression.

^b^ Number of independent transgene insertions assayed.

^c^ ANOVA, *p*-value for X-linked versus autosomal inserts.

### X Suppression Is Not Limited to Transgene Reporters

**Fig 1:**
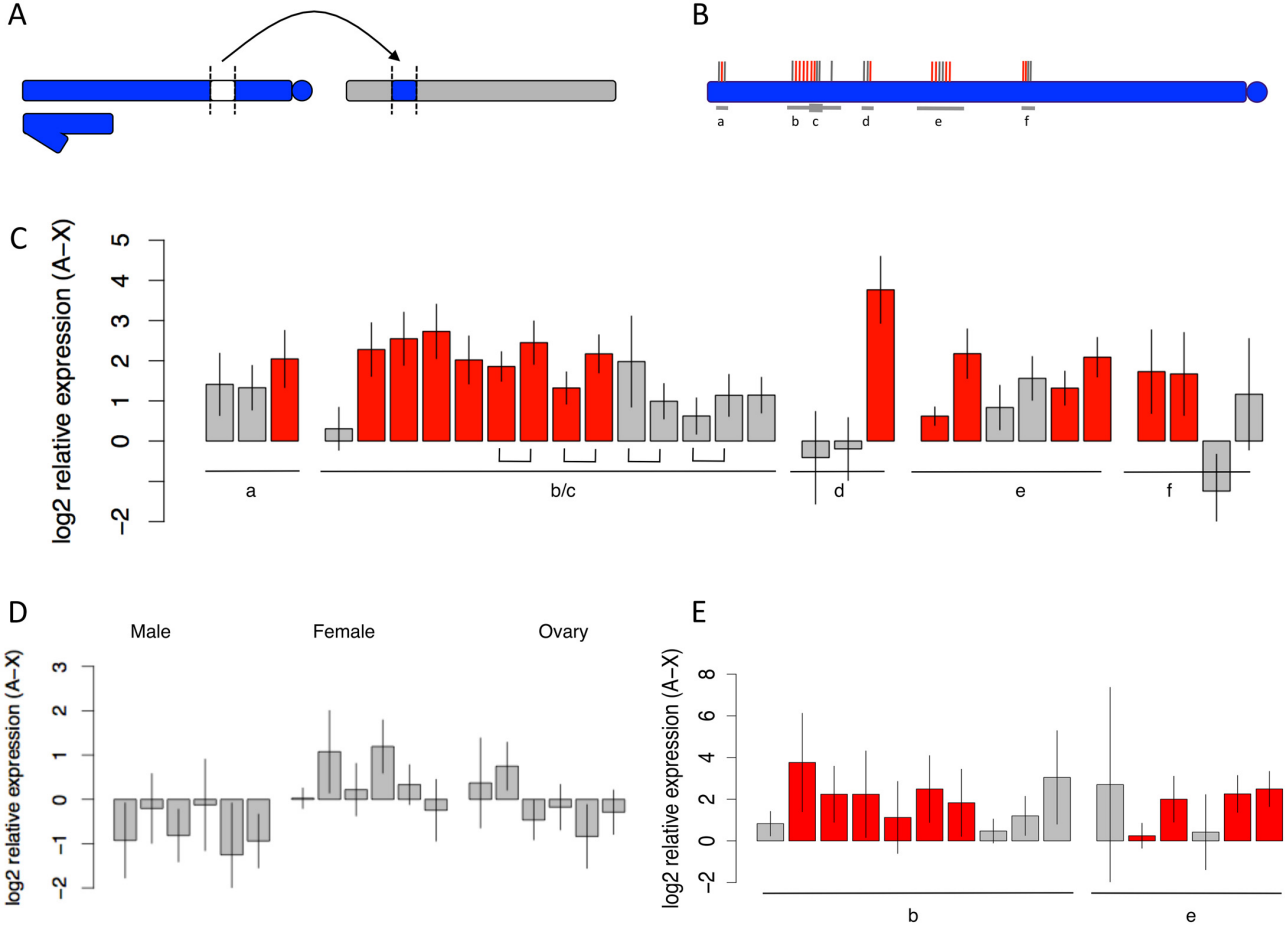
All testis-specific genes and most housekeeping genes show increased expression when moved from the X chromosome to an autosome. (A) Representative transposition genotype used to assay the effects of X-linked versus autosomal location on gene expression. X and Y chromosomes are shown in blue (the Y is smaller, hooked), and a single, representative autosomal arm is shown in gray. (B) X chromosome with wild-type locations of genes whose expression was assayed by quantitative real time PCR (qRT-PCR). Fourteen testes-specific genes and 12 housekeeping genes are indicated above the chromosome in red and gray, respectively. Two transpositions and four “synthetic” transpositions, made by combining X-linked deficiencies with autosomes bearing complementing X chromosome duplications, were used. Bars below the chromosome labeled a–f indicate the approximate sizes and locations of transposed X chromosome segments. (C) Widespread overexpression in whole testes of X-linked genes when transposed to an autosome. Testis-specific genes are indicated in red, housekeeping genes in gray. Four genes were assayed in two independent transpositions (b and c), and brackets below the barplot connect expression measurements for the same gene in different transposition genotypes. (D) No signal of release from X suppression among X-linked genes transposed to an autosome when assayed in male carcass, female carcass, or ovary. (E) Widespread overexpression of X-linked genes when transposed to an autosome in purified male germline cells with encasing somatic sheath removed. Bar height indicates the log2 difference between expression levels of testis-specific (red) and housekeeping (gray) genes when transposed to an autosome versus their endogenous X-linked location. Letters below the bars correspond to transpositions diagramed in Fig 1B. Error bars indicate 95% confidence intervals. Data found in S1 Data.

All previous studies of X suppression in the *Drosophila* male germline have involved transgene reporters embedded in transposable element vectors. It is therefore possible that X suppression is transposon-specific, reflecting an X chromosome versus autosome difference in the efficacy of transposon silencing in the male germline. To test if X suppression affects endogenous genes, we assayed expression in whole testes of genes in X chromosome segments transposed to autosomal locations. These experiments allow us to directly compare expression of endogenous X-linked genes when located on the X chromosome versus an autosome. We used two large (~2.5 Mb) transposition genotypes (e.g., *X/Y*; *Tp(1;2)/+*) and four small (~63 kb) “synthetic transposition” deficiency-duplication genotypes (e.g., *Df(1)/Y; Dp(1;3)/+*) made by combining X chromosome deficiencies with complementing X-to-autosome duplications (Fig 1A; see Materials and Methods). Importantly, gene dose is controlled in these experiments, as we contrast expression of one gene copy on the X in wild-type males with one gene copy on an autosome in males heterozygous for transpositions. In total, we assayed expression of 26 genes from two large transpositions and four small synthetic transpositions, with transposed X chromosome segments ranging in size from 2.55 Mb (*Tp(1;2)rb*^*+*^*71g*) to 63 kb (*Dp(1;3)DC523*) (Table 2). Notably, the *Tp(1;2)sn+72d* and *Tp(1;2)rb*^*+*^*71g* transpositions each include genes—*CG10920*, *CG12681* and *Act5C*—whose promoters show evidence of X suppression in previous transgene reporter assays [53].

**Table 2:** Testis expression of X-linked genes in wild-type versus transposition males.

Gene	Testis-specific^a^	*Tp(X;A)* genotype	Genome Coordinate	X-linked^b^	SE	*Tp*(*X;A*) location	Autosomal^b^	SE	*Tp(X;A)-WT*	A/X	*p*-Value^c^
*cin*	N	*Df(1)BSC843/Y; Dp(1;3)DC004/+*	255,674..258,469	-6.521	0.074	65B2	-5.110	0.281	1.411	**2.66**	**5.96E-03**
*CG13377*	N	*Df(1)BSC843/Y; Dp(1;3)DC004/+*	258,335..263,999	-4.745	0.121	65B2	-3.418	0.196	1.327	**2.51**	**8.26E-04**
*CG12470*	Y	*Df(1)BSC843/Y; Dp(1;3)DC004/+*	307,684..308,935	4.283	0.090	65B2	6.328	0.259	2.044	**4.12**	**7.15E-04**
*Vap33*	N	*Tp(1;2)rb*^*+*^*71g/Y*	3,948,975..3,955,442	-4.466	0.194	23A1-23A5	-4.160	0.077	0.306	**1.24**	0.200
*CG15572*	Y	*Tp(1;2)rb*^*+*^*71g/Y*	4,268,701..4,271,083	-1.535	0.242	23A1-23A5	0.743	0.116	2.278	**4.85**	**1.82E-04**
*CG15578*	Y	*Tp(1;2)rb*^*+*^*71g/Y*	4,277,547..4,277,961	-1.750	0.185	23A1-23A5	0.797	0.215	2.547	**5.84**	**2.14E-05**
*CG12681*	Y	*Tp(1;2)rb*^*+*^*71g/Y*	4,875,670..4,877,263	0.430	0.216	23A1-23A5	3.159	0.040	2.729	**6.63**	**8.00E-04**
*CG5062*	Y	*Tp(1;2)rb*^*+*^*71g/Y*	5,180,182..5,182,369	0.632	0.213	23A1-23A5	2.651	0.121	2.019	**4.05**	**1.30E-04**
*CG3323*	Y	*Tp(1;2)rb*^*+*^*71g/Y*	5,302,988..5,305,691	2.686	0.193	23A1-23A5	5.136	0.108	2.450	**5.46**	**2.36E-05**
*CG17764*	Y	*Tp(1;2)rb*^*+*^*71g/Y*	5,306,261..5,307,417	1.195	0.171	23A1-23A5	3.365	0.082	2.171	**4.50**	**3.66E-05**
*snf*	N	*Tp(1;2)rb*^*+*^*71g/Y*	5,309,242..5,310,501	0.883	0.151	23A1-23A5	1.873	0.108	0.990	**1.99**	**9.661E-04**
*Rnp4F*	N	*Tp(1;2)rb*^*+*^*71g/Y*	5,318,928..5,322,696	-1.259	0.190	23A1-23A5	-0.123	0.068	1.137	**2.20**	**2.42E-03**
*Act5C*	N	*Tp(1;2)rb*^*+*^*71g/Y*	5,900,861..5,905,399	0.585	0.100	23A1-23A5	1.728	0.155	1.143	**2.21**	**4.96E-04**
*CG3323*	Y	*Df(1)BSC823/Y; Dp(1;3)DC130/+*	5,302,988..5,305,691	3.527	0.126	65B2	5.384	0.089	1.857	**3.62**	**5.21E-06**
*CG17764*	Y	*Df(1)BSC823/Y; Dp(1;3)DC130/+*	5,306,261..5,307,417	2.428	0.077	65B2	3.749	0.143	1.321	**2.50**	**1.66E-04**
*snf*	N	*Df(1)BSC823/Y; Dp(1;3)DC130/+*	5,309,242..5,310,501	-2.980	0.412	65B2	-1.000	0.115	1.980	**3.94**	**6.96E-03**
*Rnp4F*	N	*Df(1)BSC823/Y; Dp(1;3)DC130/+*	5,318,928..5,322,696	-0.790	0.090	65B2	-0.170	0.161	0.620	**1.54**	**1.40E-02**
*CG3198*	N	*Df(1)BSC535/Y; Dp(1;3)DC026/+*	6,668,539..6,673,973	-2.164	0.300	65B2	-2.575	0.379	-0.411	**0.75**	0.423
*CG3192*	N	*Df(1)BSC535/Y; Dp(1;3)DC026/+*	6,677,262..6,679,118	1.201	0.259	65B2	1.006	0.157	-0.195	**0.87**	0.547
*CG4095*	Y	*Df(1)BSC535/Y; Dp(1;3)DC026/+*	6,681,767..6,683,696	-5.173	0.249	65B2	-1.407	0.244	3.766	**13.61**	**1.55E-05**
*CG11369*	Y	*Tp(1;2)sn*^*+*^*72`/Y*	7,574,290..7,576,767	1.563	0.067	58E	2.183	0.067	0.620	**1.54**	**3.47E-04**
*CG12689*	Y	*Tp(1;2)sn*^*+*^*72`/Y*	7,585,303..7,586,131	3.185	0.224	58E	5.360	0.065	2.176	**4.52**	**3.43E-04**
*Rab39*	N	*Tp(1;2)sn*^*+*^*72`/Y*	7,734,923..7,736,756	-4.769	0.194	58E	-3.936	0.118	0.834	**1.78**	**9.51E-03**
*Tom40*	N	*Tp(1;2)sn*^*+*^*72`/Y*	7,736,893..7,739,978	0.009	0.191	58E	1.569	0.115	1.560	**2.95**	**3.43E-04**
*CG32718*	Y	*Tp(1;2)sn*^*+*^*72`/Y*	7,778,205..7,779,370	1.091	0.105	58E	2.409	0.135	1.317	**2.49**	**2.39E-04**
*CG10920*	Y	*Tp(1;2)sn*^*+*^*72`/Y*	7,852,481..7,854,244	0.457	0.163	58E	2.545	0.125	2.088	**4.25**	**2.13E-05**
*CG8509*	Y	*Df(1)Exel6251/Y; Dp(1;3)DC523/+*	15,809,555..15,810,947	-0.468	0.302	65B2	1.262	0.332	1.730	**3.32**	**4.93E-03**
*CG8565*	Y	*Df(1)Exel6251/Y; Dp(1;3)DC523/+*	15,837,830..15,841,160	3.118	0.278	65B2	4.790	0.396	1.672	**3.19**	**5.86E-03**
*CG8578*	N	*Df(1)Exel6251/Y; Dp(1;3)DC523/+*	15,844,476..15,847,858	0.469	0.220	65B2	-0.769	0.398	-1.238	**0.42**	0.033*
*UBL3*	N	*Df(1)Exel6251/Y; Dp(1;3)DC523/+*	15,849,731..15,853,175	-1.526	0.432	65B2	-0.360	0.415	1.166	**2.24**	0.0877

^a^ Genes with tissue specificity of *τ* > 0.8 and maximum expression in testes.

^b^ Log2 expression relative to control gene expression.

^c^ *p*-values from paired *t* tests.

* Significant expression change in alternative direction, X>A.

We find that 23 of 26 (85%) X chromosome genes have higher expression when transposed to an autosome with an average 3.69-fold increase in expression (Wilcoxon signed-rank test, *p* = 1.82 x 10^−6^; Table 2). Twenty-one of twenty-six (81%) genes, including *CG10920*, *CG12681*, and *Act5C*, have significantly higher expression when transposed to an autosome, with individually significant transposed genes showing an average 3.85-fold increase in expression. Only one X-linked gene shows significantly lower expression when transposed to an autosome (*CG8758*; Table 2). These results recapitulate and extend findings from the transgene reporter assays and show that X suppression is not limited to genes in transposon vectors. Furthermore, we find no difference in the magnitude of escape from X suppression for small (~63 kb) versus large (~2.55 Mb) transpositions (unpaired *t* test, *p* = 0.610) suggesting that X suppression does not depend on the size of the transposition. To further test the effect of chromosomal scale on X suppression, we compared the magnitude of escape from X suppression for four genes (*CG17764*, *CG3323*, *snf*, *Rnp4f*) when in either a small versus large transposition and again found no difference (paired *t* test, *p* = 0.421). These results show that X suppression holds across multiple transpositions that vary in size and genomic location.

We next examined the effect of testis-specificity on X suppression by comparing whole testes expression of 14 testis-specific genes versus 12 non-specific genes across the six transposition genotypes (Table 2). All 14 testis-specific genes and 8 of 12 non-specific genes are significantly overexpressed when transposed from X to autosome (Table 2 and Fig 1C). Transposed testis-specific genes show an average 4.66-fold increased expression, whereas transposed non-specific genes show an average 1.95-fold increased expression (Mann-Whitney test, *P*_MWU_ < 2.2e^-16^). Among transposed genes with individually significant over-expression, non-specific genes show an average 2.42-fold increase in expression.

**Fig 2:**
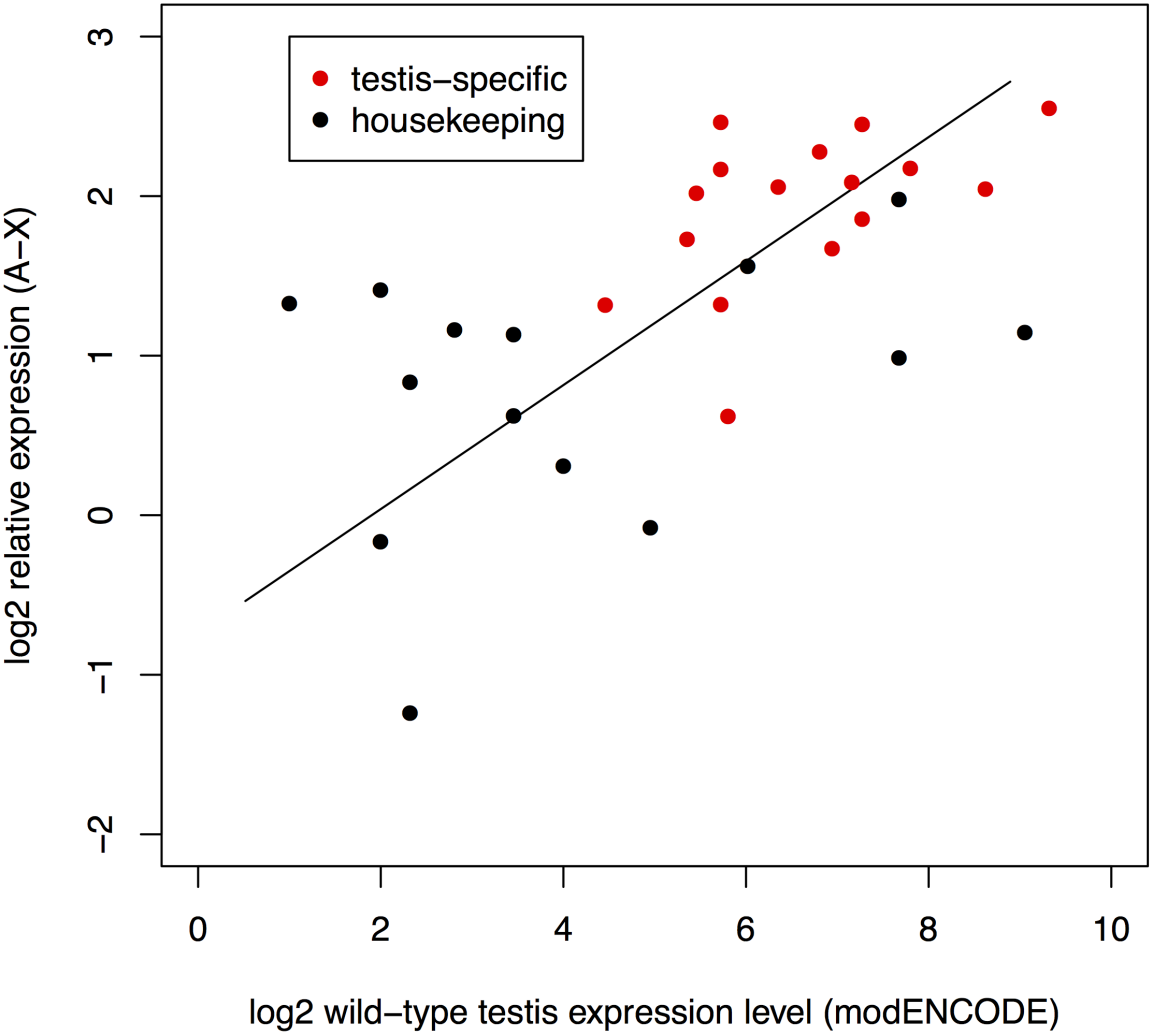
X-linked genes with higher expression levels in wild-type testes show a greater magnitude of escape from X suppression when relocated to an autosome. Standard major axis regression of magnitude of X suppression and expression level determined from RNAseq analysis of dissected testes reveals a significant relationship (*r*^2^ = 0.36, *p* = 0.0005). It is unclear whether the nature or magnitude of this relationship differs between testis-specific and housekeeping genes; this regression is non-significant within housekeeping genes (*p* = 0.22) and marginally significant within testis-specific genes (*r*^2^ = 0.26, *p* = 0.05), but there is no significant difference in the regression slope estimate between these two groups (*p* = 0.66, by likelihood ratio test using the smatr package in R). Data found in S1 Data.

While suggestive that endogenous X-linked testis-specific genes may be more strongly suppressed (~4-fold) than non-specific genes (~2-fold), there is an alternative possibility. In particular, testis-specific genes tend to be strongly expressed in testes. We therefore asked if the magnitude of wild-type gene expression is predictive of the magnitude of escape from X suppression. We find that testis expression in *Tp(X;A)* males is significantly correlated with endogenous wild-type X-linked expression for all genes (*r*^*2*^ = 0.36, *p* = 0.0005, Fig 2). This relationship is not significant within housekeeping genes (*p* = 0.2) and only marginally significant within testis-specific genes (*r*^*2*^ = 0.26, *p* = 0.05), although there is no significant difference in the regression slope estimate between these two groups, (*p* = 0.66; Fig 2). These results suggest that the magnitude of escape from X suppression for the testis-specific genes assayed is greater owing to their higher endogenous wild-type expression levels in testes compared to the non-specific genes assayed. Genes with higher expression in testis may simply show a comparably greater release from X suppression when transposed to an autosome.

### X Suppression Is Male Germline-Specific

To determine if X suppression is limited to the male germline or occurs in other tissues, we tested for evidence of escape from X suppression in the female germline and in gonadectomized male and female carcass. First, we assayed expression of a transgene reporter gene driven by one of the *Ubi* promoters previously assayed in whole testes. We find no evidence of X suppression in these samples (S4 Table). Moreover, the X-linked inserts show higher expression compared to the autosomal inserts in the female and male carcass, which the opposite direction expected if X suppression is acting in the soma. Second, we assayed expression of six non-specific genes from the two large *X/Y*; *Tp(1;2)/+* genotypes and wild-type controls. We find little evidence for X suppression in these samples (Fig 1D and S1–S3 Tables). None of the X-linked genes is overexpressed when transposed to an autosome in male carcass, whereas two are significantly overexpressed in female carcass (Fig 1D and S1 and S2 Tables). In ovaries, one X-linked gene is overexpressed when transposed to an autosome, and two are significantly underexpressed (Fig 1D and S3 Table). These findings suggest X suppression is limited to gene expression in testes.

As the epithelial cells of the testis sheath are somatic, it remains possible that X suppression acts in these cells and perhaps to a lesser degree in the male germline cells. Previous gene expression analyses from male germline samples with testis sheath dissected away [40] and previous RNA in situ analyses [53] suggest that X suppression is germline-specific. We nevertheless assayed expression from 16 endogenous genes (10 testis-specific, 6 non-specific) from the two large *X/Y*; *Tp(1;2)/+* genotypes in purified male germline samples with testes sheaths removed (see Materials and Methods**)** [42,50]. We find that 13/16 genes show significant evidence of escape from X suppression, with non-specific and testis-specific genes showing 3.34- and 5.0-fold average increases in expression, respectively (one-sample *t* test, *p* = 0.034 and *p* = 0.0011, respectively; Fig 1E and Table 3). These findings strongly suggest that X suppression is specific to the *Drosophila* male germline.

**Table 3:** Expression of X-linked genes in wild-type versus transposition male germline cells.

Gene	Testis-specific^a^	*Tp(X;A)* genotype	Genome Coordinate	X-linked^b^	SE	*Tp*(*X;A*) location	Autosomal^b^	SE	*Tp(X;A)-WT*	A/X	*p-*Value^c^
*Vap33*	N	*Tp(1;2)rb*^*+*^*71g/Y*	3,948,975..3,955,442	-7.896	0.194	23A1-23A5	-7.070	0.165	0.826	1.77	**1.23E-02**
*CG15572*	Y	*Tp(1;2)rb*^*+*^*71g/Y*	4,268,701..4,271,083	-5.243	0.148	23A1-23A5	-1.481	0.859	3.762	13.567	**1.10E-02**
*CG15578*	Y	*Tp(1;2)rb*^*+*^*71g/Y*	4,277,547..4,277,961	-3.002	0.347	23A1-23A5	-0.762	0.460	2.240	4.725	**5.31E-03**
*CG12681*	Y	*Tp(1;2)rb*^*+*^*71g/Y*	4,875,670..4,877,263	1.512	0.756	23A1-23A5	3.750	0.159	2.239	4.720	**3.99E-02**
*CG5062*	Y	*Tp(1;2)rb*^*+*^*71g/Y*	5,180,182..5,182,369	0.799	0.629	23A1-23A5	1.925	0.311	1.126	2.182	**1.61E-01**
*CG3323*	Y	*Tp(1;2)rb*^*+*^*71g/Y*	5,302,988..5,305,691	1.831	0.521	23A1-23A5	4.321	0.459	2.490	5.618	**7.28E-03**
*CG17764*	Y	*Tp(1;2)rb*^*+*^*71g/Y*	5,306,261..5,307,417	2.512	0.583	23A1-23A5	4.342	0.071	1.829	3.554	**3.44E-02**
*snf*	N	*Tp(1;2)rb*^*+*^*71g/Y*	5,309,242..5,310,501	0.387	0.148	23A1-23A5	0.861	0.122	0.474	1.39	**4.01E-02**
*Rnp4F*	N	*Tp(1;2)rb*^*+*^*71g/Y*	5,318,928..5,322,696	-2.756	0.085	23A1-23A5	-1.556	0.344	1.200	2.298	**2.30E-02**
*Act5C*	N	*Tp(1;2)rb*^*+*^*71g/Y*	5,900,861..5,905,399	-5.529	1.210	23A1-23A5	-2.828	0.201	2.316	4.980	0.128
*CG11369*	Y	*Tp(1;2)sn*^*+*^*72`/Y*	7,574,290..7,576,767	2.243	0.203	58E	2.492	0.159	0.248	1.188	0.165
*CG12689*	Y	*Tp(1;2)sn*^*+*^*72`/Y*	7,585,303..7,586,131	4.574	0.375	58E	6.577	0.287	2.002	4.006	**3.29E-03**
*Rab39*	N	*Tp(1;2)sn*^*+*^*72`/Y*	7,734,923..7,736,756	-9.634	0.410	58E	-9.210	0.411	0.424	1.341	0.563
*Tom40*	N	*Tp(1;2)sn*^*+*^*72`/Y*	7,736,893..7,739,978	-0.214	0.701	58E	2.831	0.674	3.045	8.254	**1.41E-02**
*CG32718*	Y	*Tp(1;2)sn*^*+*^*72`/Y*	7,778,205..7,779,370	0.188	0.266	58E	2.443	0.277	2.255	4.773	**3.77E-04**
*CG10920*	Y	*Tp(1;2)sn*^*+*^*72`/Y*	7,852,481..7,854,244	-0.139	0.205	58E	2.352	0.294	2.491	5.623	**2.03E-04**

^a^ Genes with tissue specificity of *τ* ≥ 0.8 and maximum expression in testes.

^b^ Log2 expression relative to control gene expression.

^c^ *p*-values from paired *t* tests.

### No Signal of X Suppression or Escape from X Suppression in X-Autosome Translocations

Promoter sequences in transgenes and endogenous genes in small and large transpositions can escape X suppression when moved from X-linked to autosomal locations (Fig 1 and Tables 1 and 2). To further investigate the physical scale of X suppression, we assayed expression of the same 26 genes used in the transposition experiments (Table 2) in whole testes of males bearing X-autosome reciprocal translocations. In contrast to transgenes and transpositions, X-autosome translocations are large chromosome-scale aberrations. To identify translocations, we screened all publicly available *T(1;A)* translocation stocks with known breakpoints from the Bloomington (*n* = 6) and Kyoto Stock Centers (*n* = 7) but found only two translocations—*T(1;3)OR17* and *T(1;3)l-v455—*still segregating in the stocks, the others being lost prior to receipt. For *T(1;3)OR17*, cytological divisions 1–19EF of the *X* are translocated to cytological division 67C on chromosome arm *3L*, and cytological divisions 61–67C are translocated to cytological division 19EF the *X* (S1 Fig). For *T(1;3)l-v455*, cytological divisions 1–3C are translocated to cytological division 81 on *3R*, and cytological divisions 81–100 are translocated to cytological division 3C on the *X* (S1 Fig). *T(1;3)OR17* is male-fertile, and *T(1;3)l-v455* is male-sterile.

In *T(1;3)OR17* males, all 26 X chromosome genes are translocated to *3L* (S1 Fig), but none are overexpressed relative to wild-type controls (Table 4). Instead, only five genes differ significantly from wild type, and all are *under*expressed when translocated to *3L*—the opposite pattern expected for escape from X suppression. We conclude that there is no escape from X suppression in *T(1;3)OR17* males.

**Table 4:** Testis expression of genes in *T(1;3)* translocation versus wild-type males.

				*T(1;3)OR17*						*T(1;3)l-v455*						Between translocations
	Gene	Testis-specific^a^	Position	*FM6*^b^	SE	*T(1;3)*^b^	SE	Moved?	*p-*Value^c^	*FM6*^b^	SE	*T(1;3)*^b^	SE	Moved?	*p*-Value^c^	*p*-Value^e^
1	*CG12470*	Y	1A1	2.656	0.174	2.500	0.109	Y	0.500	2.225	0.214	3.174	0.050	Y	**0.041**	**0.013**
2	*CG13377*	N	1A1	-6.386	0.340	-6.444	0.052	Y	0.880	-6.278	0.409	-6.408	0.135	Y	0.788	0.821
3	*cin*	N	1A1	-7.595	0.211	-7.675	0.079	Y	0.749	-8.297	0.166	-8.040	0.273	Y	0.474	0.312
4	*CG13359*^d^	Y	1C5	2.979	2.986	1.987	1.299	Y	0.782	1.661	1.231	2.545	2.757	Y	0.790	0.867
5	*CG14635*^d^	Y	1D1	-1.902	1.346	-1.208	0.623	Y	0.674	-1.080	0.071	-1.714	1.056	Y	0.609	0.706
6	*CG3795*^d^	Y	2B7	2.195	0.367	1.932	0.322	Y	0.620	2.795	0.016	1.669	0.661	Y	0.230	0.745
7	*CG32806*^d^	Y	2B9	2.531	3.745	3.597	1.290	Y	0.809	1.848	1.613	2.840	3.909	Y	0.831	0.868
8	*CG14806*^d^	N	2B14	-0.056	3.591	0.699	1.262	Y	0.858	-0.791	1.499	-0.096	3.568	Y	0.870	0.850
9	*CG14054*^d^	Y	2C8-2C9	-1.632	1.166	-1.611	0.255	Y	0.988	0.152	0.024	-0.021	0.540	Y	0.779	0.080
10	*egh*^d^	N	3A6	-3.268	3.875	-1.676	1.294	Y	0.728	-3.758	1.236	-3.580	4.087	Y	0.970	0.694
11	*Vap33*	N	3F9	-7.484	0.164	-7.497	0.159	Y	0.956	-7.393	0.214	-7.385	0.536	N	0.990	0.857
12	*CG15572*	Y	4B4	-3.146	0.215	-3.599	0.075	Y	0.160	-3.633	0.201	-3.293	0.394	N	0.499	0.521
13	*CG15578*	Y	4B4	-3.723	0.209	-4.078	0.281	Y	0.373	-4.697	0.512	-3.633	0.587	N	0.245	0.545
14	*CG12681*	Y	4D3	4.139	0.420	3.876	0.155	Y	0.606	4.400	0.193	3.994	0.276	N	0.302	0.735
15	*CG5062*	Y	4F2	-1.805	0.199	-1.834	0.033	Y	0.898	-1.983	0.258	-1.807	0.152	N	0.596	0.880
16	*CG17764*	Y	4F4	-1.054	0.073	-1.612	0.110	Y	0.019*	-1.404	0.220	-1.453	0.174	N	0.871	0.490
17	*CG3323*	Y	4F4	0.364	0.106	0.217	0.016	Y	0.297	0.371	0.314	0.565	0.112	N	0.610	0.088
18	*snf*	N	4F4	-1.817	0.139	-1.505	0.097	Y	0.148	-1.704	0.154	-1.691	0.081	N	0.942	0.218
19	*Rnp4F*	N	4F5	-4.388	0.127	-4.274	0.040	Y	0.469	-4.598	0.297	-4.593	0.145	N	0.989	0.152
20	*Act5C*	N	5C7	-2.232	0.535	-2.104	0.139	Y	0.836	-3.217	0.236	-2.689	0.502	N	0.415	0.364
21	*CG3198*	N	6C4	-7.005	0.256	-6.783	0.043	Y	0.478	-7.221	0.153	-7.002	0.382	N	0.636	0.626
22	*CG3192*	N	6C5	-2.298	0.053	-2.414	0.041	Y	0.163	-2.700	0.180	-2.670	0.209	N	0.920	0.345
23	*CG4095*	Y	6C5	-3.119	0.126	-3.341	0.088	Y	0.231	-3.339	0.213	-3.167	0.391	N	0.725	0.704
24	*CG11369*	Y	7B4	-0.957	0.104	-2.477	0.048	Y	0.001*	-1.222	0.276	-1.623	0.339	N	0.413	0.125
25	*CG12689*	Y	7B4	1.056	0.117	0.562	0.041	Y	0.040*	0.692	0.222	1.367	0.078	N	0.080	0.003*
26	*Rab39*	N	7B7	-7.301	0.083	-7.629	0.055	Y	0.037*	-8.021	0.074	-7.634	0.245	N	0.251	0.987
27	*Tom40*	N	7B7	-2.037	0.114	-2.413	0.070	Y	0.061	-2.505	0.187	-2.443	0.147	N	0.808	0.862
28	*CG32718*	Y	7B8	-1.399	0.144	-1.732	0.089	Y	0.135	-1.706	0.372	-1.412	0.013	N	0.512	0.066
29	*CG10920*	Y	7C1	-1.383	0.111	-1.776	0.008	Y	0.071	-1.950	0.364	-1.484	0.118	N	0.329	0.131
30	*CG8509*	Y	13F1	-1.889	0.590	-1.629	0.050	Y	0.703	-1.548	0.276	-1.545	0.110	N	0.993	0.542
31	*CG8565*	Y	13F10	1.863	0.079	1.554	0.027	Y	0.047*	1.361	0.270	1.902	0.124	N	0.173	0.102
32	*CG8578*	N	13F10	-4.559	0.164	-4.992	0.029	Y	0.114	-4.958	0.181	-5.387	0.491	N	0.482	0.505
33	*UBL3*	N	13F13	-1.700	0.067	-1.468	0.103	Y	0.143	-2.081	0.227	-1.984	0.257	N	0.791	0.172
34	*CG31528*	Y	82B1	-0.573	0.152	-0.281	0.681	N	0.713	-1.452	0.464	-1.959	0.691	Y	0.580	0.159
35	*CG31526*	Y	82C1	-2.095	0.216	-2.374	0.397	N	0.579	-2.246	0.278	-2.488	0.092	Y	0.482	0.804
36	*CG7920*	N	99D1	0.623	0.140	0.150	0.423	N	0.383	0.149	0.308	-0.822	0.567	Y	0.227	0.247
37	*ocn*	Y	99D3	3.724	0.155	3.271	0.371	N	0.351	2.805	0.302	2.648	0.577	Y	0.825	0.423
38	*janA*	N	99D3	-4.258	0.377	-3.204	0.133	N	0.095	-2.885	0.606	-3.445	0.482	Y	0.512	0.672
39	*janB*	Y	99D3	2.200	0.182	2.806	0.349	N	0.221	3.094	0.563	2.905	0.672	Y	0.840	0.904
40	*Rpl32*	N	99D8	-6.248	0.178	-6.618	0.151	N	0.189	-7.180	0.096	-6.498	0.202	Y	0.060	0.662
41	*CG31029*	Y	99D3	-0.003	0.275	-0.327	0.561	N	0.641	-1.332	0.747	-1.490	0.250	Y	0.857	0.163
42	*Tpi*	N	99D8	0.481	0.290	0.465	0.524	N	0.981	-0.480	0.409	-1.318	0.304	Y	0.181	0.055

^a^ Genes with tissue specificity of *τ* ≥ 0.8 and maximum expression in testes.

^b^ Log2 expression relative to control gene expression.

^c^ Two sample *t* test comparing expression of genes when on control chromosomes (*FM6*) versus translocation chromosomes.

^d^Additional genes assayed that were not assayed in the transposition analyses.

^e^ Two sample *t* test comparing expression of genes when on *T(1;3)l-v455* versus *T(1;3)OR17*.

* Significant expression change in alternative direction, X>A.

In *T(1;3)l-v455* males, only three of the 26 X chromosome genes are translocated to *3R* (S1 Fig). It is important to note that the amount of the X-linked material translocated to the autosome in *T(1;3)l-v455* (~3 Mb) is similar to that for largest transposition assayed (2.55 Mb in *Tp(1;2)rb*^*+*^*71g*). However, unlike the large transposition, only one gene (*CG12740*) shows a marginally significant ~1.9-fold increase in expression relative to wild-type controls when translocated to *3R* (Table 4). To further test if escape from X suppression acts in translocations, we assayed seven additional genes (five testis-specific, two non-specific) located within the X-linked region transposed to the autosome in *T(1;3)l-v455* and in *T(1;3)OR17* (S1 Fig). None differ in expression from wild-type controls for either translocation (Table 4, lines 4–10). These findings suggest that the increased expression of *CG12470* in *T(1;3)l-v455* is incidental to escape from X suppression. We therefore conclude that X-linked genes do not escape X suppression in X-autosome translocations.

We next tested if naïve autosomal genes experience X suppression when translocated to the X chromosome. We assayed an additional five testis-specific genes and four non-specific genes located on autosomal arm *3R* (Table 4). One of these testis-specific genes is *ocnus*, an autosomal gene known to undergo X suppression in transgene reporter assays [51,53]. In *T(1;3)l-v455* males, all nine genes are translocated from *3R* to the *X* (none are translocated in *T(1;3)OR17)*, but none show a significant change in expression relative to wild-type controls (Table 4). It is important to note, however, that *T(1;3)l-v455* males retain a wild-type third chromosome (S1 Fig), decreasing our power to detect reduced expression. Overall, these findings suggest that, in contrast to genes that have been relocated via transposition or trangenesis, translocated X chromosome genes do not escape X suppression and translocated autosomal genes show little evidence of X suppression.

### Is Putative MSCI Disrupted in X-Autosome Translocations?

Assaying gene expression from fertile and sterile X-autosome translocations allows us to test for another form of X chromosome regulation hypothesized to act in the male germline: MSCI. The male sterility of ~75% of X-autosome translocations has been interpreted as evidence that these chromosome rearrangements disrupt MSCI [50]. In particular, X-autosome translocations could disrupt MSCI in two ways: X-linked genes translocated to an autosome could escape MSCI, resulting in their aberrant overexpression [50]; or, autosomal genes translocated to the X could be transcriptionally silenced by MSCI, resulting in their aberrant underexpression (as in mouse; [27,55,56]). We tested both possibilities. First, we compared the expression of ten X chromosome genes translocated to chromosome *3* in testes from *T(1;3)l-v455* males, which are sterile, versus *T(1;3)OR17* males, which are fertile. Only one of the genes shows a significant increase in expression in *T(1;3)l-v455* males compared to *T(1;3)OR17* males when translocated to chromosome *3* (*CG12470*; *p* = 0.013, Table 4). Second, we compared testis expression of eight autosomal genes that are translocated to the *X* in *T(1;3)l-v455* males (sterile) but remain autosomal in *T(1;3)OR17* males (fertile). None of the eight autosomal genes show a significant decrease in expression when translocated to the X in male-sterile *T(1;3)l-v455* flies (Table 4). These results show that any putative MSCI in the *Drosophila* male germline does not appear to be disrupted in a way that results in aberrant transcriptional expression of genes translocated between the X chromosome and autosomes. Alternatively, the effects of MSCI could be too subtle to detect via our whole testis dissections [49]. We note, however, that whole testis dissections are easily sufficient to detect X suppression (and escape from X suppression) using transgenes and transpositions (Tables 1 and 2; [40,51,53,57]).

### X Chromosome Recruitment of Compensatory, Testis-Specific Promoter Elements

**Fig 3:**
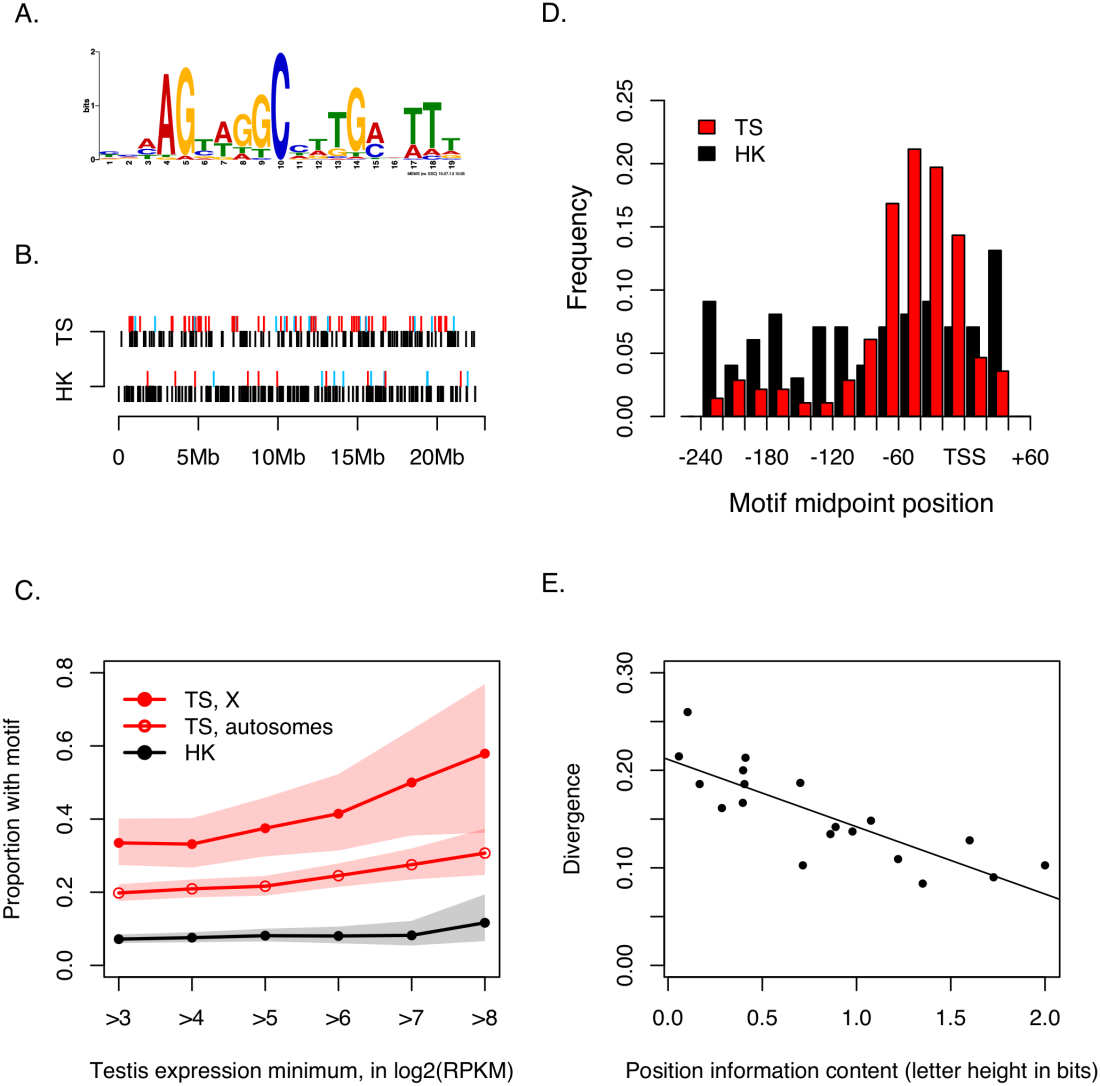
An evolutionarily conserved promoter element compensates X suppression. (A) MEME-generated logo plot of the AG[tagg]C motif. (B) AG[tagg]C motif instances in the (-250 bp, + 50 bp) upstream regions of testis-specific (“TS”) and housekeeping (“HK”) genes along the X chromosome. In both diagrams, a red line indicates presence of the motif in the coding orientation, a blue line presence of the motif in the reverse orientation, and a black line absence of the motif for a particular gene. (C) Proportion of genes containing a coding-orientation AG[tagg]C motif in the (-250 bp, +50 bp) upstream regions in subsets of testis-specific and housekeeping genes having a minimum level of testis expression. Shaded areas indicate 95% CIs for the observed proportions. (D) Histograms of the positions relative to transcription start sites (TSS) of the 279 coding-orientation AG[tagg]C motifs found in testis-specific genes (red) and the 99 found in housekeeping genes (black). (E) Linear regression (*y* = -0.69*x* + 0.21, *R*^2^ = 0.6755, *p* = 1.59 x 10^−5^) of divergence between *D*. *melanogaster* and *D*. *yakuba* on total “bit height” (see panel A) of each (coding-oriented) motif position, for testis-specific genes. The corresponding regression using housekeeping genes is not significant (*p* = 0.588; S3 Fig). Data found in S2 Data.

From the transgene and transposition experiments, we infer that transcription from the X chromosome is 2- to 4-fold suppressed in the male germline. And yet, in the testes of wild-type males, global germline expression levels from the X chromosome are *not* ~2- to 4-fold lower than that from the autosomes [40,42], implying that X suppression is compensated. We speculated that transcription from the X is suppressed in the male germline but that X-linked testes-specific genes may have evolved strong promoters that counteract suppression. We tested the possibility that promoters of testis-specific X-linked genes might have recruited particular sequence elements that drive strong expression. Using the MEME motif-discovery software [58], we computationally queried sequence coordinates from -250 bp upstream to +50 bp downstream of the transcription start sites (TSS) of subsets of genes in the *D*. *melanogaster* reference genome (see Materials and Methods; S2 Fig). In our query of testis-specific X-linked genes, we identified eight DNA sequence motifs, one of which was significantly enriched in promoter regions of testis-specific genes compared to housekeeping genes (Fisher’s exact *P*_FET_ < 2.2 x 10^−16^; S5 Table). This ~19-bp sequence (hereafter “AG[tagg]C”, based on the seven least-degenerate core nucleotides within the sequence) has a complex sequence, is abundant (being present in the promoter regions of 1,189 genes (7.8%; Fig 3A and Table 5) and not only shows a 3.5-fold enrichment at genes with testis-specific expression versus housekeeping genes (S5 Table) but, among testis-specific genes, is 2-fold overrepresented on the X chromosome relative to autosomes (*P*_FET_ < 1.04 x 10^−5^; Fig 3B and 3C and Table 5). Indeed, the X chromosome enrichment increases with expression level in testis (Fig 3C). The greatest X-autosome disparity occurs for the most strongly expressed testis-specific genes, peaking at 31% of autosomal genes versus 58% of X-linked genes (Fig 3C). In contrast, the AG[tagg]C motif is found upstream of just 4.8% to 7.6% of X-linked and autosomal housekeeping genes and non-testis tissue-specific genes (Table 5). As might be expected given its enrichment at testis-specific genes, the AG[tagg]C motif shows no similarity to the GA-motif that mediates somatic sex chromosome dosage compensation via recruitment of the MSL complex [20].

**Table 5:** The AG[tagg]C motif is enriched in the promoters of testis-specific genes on the X chromosome.

	X chromosome			Autosomes			OR^b^	FET *p*-Value^c^
	≥1 motif	total	ppn^a^	≥1 motif	total	ppn^a^		
All genes	210	2315	0.091	979	12842	0.076	1.21	**0.019**
Housekeeping genes^d^	19	399	0.048	152	2000	0.076	0.6	**0.043**
Tissue-specific (non-testis) genes^e^	26	361	0.072	145	2078	0.070	1.1	0.649
Testis-specific genes^f^	78	241	0.324	253	1324	0.191	2.02	**1.04E-05**

^a^ Proportion genes with ≥1 AG[tagg]C motif

^b^ Odds-ratio for motif presence on X versus autosome

^c^ Fisher's exact probability

^d^ Genes having tissue specificity of *τ* ≤ 0.2 (see Methods).

^e^ Genes having tissue specificity of *τ* ≥ 0.8, with maximum expression in any tissue other than testes.

^f^ Genes having tissue specificity of *τ* ≥ 0.8, with maximum expression in testes.

Being enriched in the upstream regions of X-linked testis-specific genes, the AG[tagg]C motif is a plausible candidate promoter element that might mediate the strength and/or specificity of testis expression. Several statistical analyses provide further support for its functional significance. First, testis-specific genes with at least one copy of AG[tagg]C have ~1.8-fold higher median expression than those lacking AG[tagg]C (*P*_MWU_ *=* 2.2 x 10^−9^); however, housekeeping genes with or without AG[tagg]C show no difference in expression (*P*_MW_ *=* 0.144). Second, the AG[tagg]C element shows DNA strand bias in testis-specific genes: 80% of single-copy AG[tagg]C motifs are found on the coding strand and 20% on the template strand (binomial test *p* < 2.2 x 10^−16^); in contrast, housekeeping genes show no such bias (*p* = 0.158). This strand bias is associated with significant differences in expression among testis-specific genes (Kruskal-Wallis, *P*_KW_ = 3.1 x 10^−8^): those with the AG[tagg]C motif on the coding strand have 1.9-fold higher expression than those lacking the motif (*P*_MW_ = 3.0 x 10^−8^), whereas those with the AG[tagg]C motif on the template strand do not differ in expression from those lacking the motif (*P*_MW_ = 0.070). There is no evidence that AG[tagg]C presence or orientation affects the expression of housekeeping genes (*P*_KW_ = 0.059). Third, within the 300-bp promoter regions queried, AG[tagg]C motif locations are concentrated about a modal position centered at -40 bp upstream of the TSSs of testis-specific genes (*χ*^2^ goodness-of-fit compared to a uniform distribution, *p* < 2.2 x 10^−16^); in contrast, the AG[tagg]C motif location shows no strong pattern in housekeeping genes (*p* = 0.574; Fig 3D).

Finally, if AG[tagg]C is indeed important for wild-type function, then we should find evidence of functional constraints in its DNA sequence evolution. As AG[tagg]C is repeated many times in the genome, nucleotide bit height in the logo plot (a measure of nucleotide frequency at a particular site) provides quantitative information on the relative importance of particular nucleotides to motif function (Fig 3A). We therefore examined DNA sequence divergence of homologous AG[tagg]C elements on coding-strands between *D*. *melanogaster* and its related species, *D*. *yakuba*, for both testis-specific genes and housekeeping genes. We find that AG[tagg]C nucleotide bit height is negatively correlated with interspecific divergence for testis specific genes (*R*^2^ = 0.676, *p* = 1.6 x 10^−5^; Fig 2E) but not housekeeping genes (*p* = 0.588; S3 Fig). For testis genes, the least degenerate nucleotide positions in AG[tagg]C are also the most evolutionarily constrained. Taken together, these findings on abundance and strand bias (Fig 3B and Table 5), expression level (Fig 3C), position relative to TSS (Fig 3D), and evolutionary constraint (Fig 3E) strongly imply that the AG[tagg]C promoter element is functional and important for expression of testis-specific, but not housekeeping, genes in the male germline.

To functionally validate the AG[tagg]C motif, we compared expression of a *lacZ* reporter driven either by wild-type or mutant AG[tagg]C sequences. We cloned the upstream noncoding sequence from *CG12681*, a testis-specific gene on the X chromosome. We found that *CG12681* escapes X suppression when transposed to an autosomal location (Table 2), and previous work showed that the *CG12681* promoter region drives strong *lacZ* reporter gene expression in testes and escapes from X suppression when moved to an autosomal site via transgene [53]. We therefore cloned the identical 766-bp upstream noncoding region of *CG12681* [53] which we determined includes two copies of the AG[tagg]C motif, –193 bp and –51 bp upstream of the TSS. After cloning the wild-type sequence, we used site-directed mutagenesis to generate lesions that alter the proximal AG[tagg]C element, the distal AG[tagg]C element, or both (Table 6, column 2; Materials and Methods; S4 Fig). The wild-type AG[tagg]C sequence and the four mutant AG[tagg]C sequences were then separately cloned upstream of the *lacZ* reporter gene, and the resulting AG[tagg]C-*lacZ* sequences subcloned into the *attB* vector [59], yielding *attB{w*^*+*^; AG[tagg]C-*lacZ}* (Materials and Methods; S4 Fig). We then generated ten transgenic genotypes: each of the five *attB{w*^*+*^; AG[tagg]C-*lacZ}* constructs (wild type and the four mutant AG[tagg]C motifs) in a common X chromosome *attP* insertion landing site at cytological position 5B8; and each of the five *attB{w*^*+*^*;* AG[tagg]C-*lacZ}* constructs into a common autosomal *attP* landing site at cytological position 75A10 on arm *3L*. Finally, we assayed *lacZ* expression by qRT-PCR to determine if, and to what extent, mutations in the AG[tagg]C motifs affect gene expression from the X-linked versus third chromosome insertion sites.

**Table 6:** Expression of wild-type and experimentally altered AG[tagg]C motifs in testes.

		X-linked^b^				Autosomal^b^				
Transgene	Motif sequences^a^	Expression^c^	SE	Fold-change	*P*_*WT-X*_^d^	Expression^c^	SE	Fold-change	*P*_*WT-A*_^d^	*P*_*X-A*_^e^
WT	AAGG**G**AAAACTT…AAGTGGG**C**CGTG	1.873	0.149	—	—	3.384	0.154		—	**0.0004**
B2	AAGG**G**AAAACTT…AAGTGGGAAGTG	0.231	0.278	-3.1	**0.0044**	2.363	0.291	-2.0	**0.0304**	**0.0018**
B5	AAGG**G**AAAACTT…ACTTGGAAAGTG	0.469	0.534	-2.6	0.3154	1.222	0.445	-4.5	**0.0045**	0.3220
A5	AAGTTTAAAAGT…AAGTGGG**C**CGTG	0.262	0.589	-3.1	0.0678	3.819	0.117	0.7	0.0685	**0.0077**
AB	AAGTTTAAAAGT…ACTTGGAAAGTG	-0.733	0.437	-6.1	**0.0062**	1.603	0.180	-3.4	**0.0003**	**0.0079**

^a^ *CG12681* has two AG[tagg]C motifs, –193 bp and –51 bp upstream of the TSS, shown here separated by "…"; nucleotides changed by site-directed mutagenesis are shown in red font.

^b^ X-linked and autosomal *attP* sites are at cytological/genomic positions 5B8/X:5,757,560 and 75A10/3L:17,952,108, respectively.

^c^ Log2 expression relative to control gene expression.

^d^ Two sample *t* test comparing reporter expression driven by wild-type motif versus experimentally alterred motifs.

^e^ Two sample *t* test comparing reporter gene expression driven from X-linked versus autosomal insertions.

Wild-type AG[tagg]C-bearing transgenes show 2.9-fold higher expression from the autosomal site than the X chromosome site (Table 6), recapitulating the escape from X suppression observed in transposition and in previous transgene genotypes [53]. For X-linked insertions, mutant AG[tagg]C sequences have 2.6- to 6.1-fold lower expression relative to wild-type controls, two significantly so and one marginally (Table 6). For autosomal insertions, three of four mutant promoters have significant 2.0- to 4.5-fold lower expression (Table 6). These findings show that both the distal and the proximal AG[tagg]C motif sequences of the *CG12681* promoter region contribute to strong expression in testis from both X-linked and autosomal sites. Notably, relative to the disrupted AG[tagg]C sequences, the X-linked wild-type AG[tagg]C promoter element provides, on average, a ~3.7-fold boost to expression, the approximate magnitude increase required to compensate for X suppression (see above). Indeed, the expression level achieved by the wild-type promoter at the X-linked insert is comparable to that achieved by mutant promoters at the autosomal site. Put differently, X and autosomal expression levels are comparable when suppression of the X-linked copy is offset by a wild-type AG[tagg]C motif. These general quantitative conclusions are, however, provisional as we have only surveyed expression from a single X-linked site and a single autosomal site.

We next asked if the AG[tagg]C sequences contribute to testis specificity *per se*, as opposed to overall testis expression level: if the AG[tagg]C motif mediates testis specificity, then disruption of the motif could yield less specific, aberrantly broad expression. To test if mutations in the AG[tagg]C motif compromise testis specificity, we compared wild-type versus mutant transgene expression in gonadectomized male carcasses. Among the autosomal insertions, all four mutant AG[tagg]C sequences drive significantly lower expression in the male carcass relative to wild type (on average, ~2.8-fold lower; Table 7). For this autosomal site, then, disrupting the AG[tagg]C motif reduces expression in testis *and* in the rest of the male carcass. The X-linked AG[tagg]C transgenes behave qualitatively differently. For the X-linked insertions, all four mutant AG[tagg]C sequences drive *higher* expression in the male carcass (on average, ~8.3-fold higher), three significantly so (on average, ~9.9-fold higher; Table 7). At the X-linked site, then, disrupting the AG[tagg]C motif reduces expression in testis but *increases* expression in the male carcass. These findings show that the AG[tagg]C motif contributes to strong testis expression and, for the X-linked site, to testis-specificity.

**Table 7:** Expression of wild-type and experimentally alterred AG[tagg]C motifs in gonadectomized males.

		X-linked^b^				Autosomal^b^				
Transgene	Motif sequences^a^	Expression^c^	SE	Fold-change	*P*_*WT-X*_^d^	Expression^c^	SE	Fold-change	*P*_*WT-A*_^d^	*P*_*X-A*_^e^
WT	AAGG**G**AAAACTT…AAGTGGG**C**CGTG	-7.339	0.758		—	-3.830	0.117		—	0.0177
B2	AAGG**G**AAAACTT…AAGTGGGAAGTG	-5.602	0.105	3.33	0.1047	-5.426	0.242	-0.33	**0.003**	0.5407
B5	AAGG**G**AAAACTT…ACTTGGAAAGTG	-4.031	0.257	9.90	**0.0172**	-6.573	0.322	-6.70	**0.002**	**0.0010**
A5	AAGTTTAAAAGT…AAGTGGG**C**CGTG	-3.434	0.114	14.99	**0.0131**	-4.790	0.273	-1.95	**0.031**	**0.0100**
AB	AAGTTTAAAAGT…ACTTGGAAAGTG	-5.044	0.346	4.91	**0.0445**	-4.975	0.384	-2.21	**0.045**	0.8986

^a^ *CG12681* has two AG[tagg]C motifs, –193 bp and –51 bp upstream of the TSS, shown here separated by "…"; nucleotides changed by site-directed mutagenesis are shown in red font.

^b^ X-linked and autosomal *attP* sites are at cytological/genomic positions 5B8/X:5,757,560 and 75A10/3L:17,952,108, respectively.

^c^ Log2 expression relative to control gene expression.

^d^ Two sample *t* test comparing reporter expression driven by wild-type motif versus experimentally alterred motifs.

^e^ Two sample *t* test comparing reporter gene expression driven from X-linked versus autosomal insertions.

## Discussion

The findings reported here lead to several conclusions concerning gene expression from the X chromosome in the *Drosophila* male germline. First, genes on the X chromosome are transcriptionally suppressed several-fold: both transgene reporters and endogenous genes show 2- to 4-fold higher expression when moved from the transcriptionally repressive environment of the X chromosome to the more permissive environment of the autosomes. Second, testis-specific genes experience larger, more consistent increases in expression than non-specific genes when moved from X-linked to autosomal positions. Preliminary evidence suggests, however, that this may be mediated by the higher absolute wild-type expression of testis-specific genes in the testes (Fig 2) rather than testis-specificity per se. Third, the AG[tagg]C motif is enriched in the upstream promoter regions of testis-specific genes, especially those on the X chromosome. The AG[tagg]C motif is evolutionarily conserved at critical nucleotide positions, drives higher average expression in testis, and, when X-linked, may contribute to testis-specificity. While the AG[tagg]C element can compensate for X suppression, we identified several other motifs with overrepresentation on the X chromosome (S1 Table), suggesting that other promoter sequences may also compensate for X suppression. Overall these findings show that expression of X-linked genes in the *Drosophila* male germline results from a balance between chromosome-wide transcriptional suppression and the evolution of strong, compensatory promoters.

The mechanism of X suppression remains unknown. It is clear that X suppression is not mediated by the promoter sequences of X-linked genes, as identical promoters drive systematically different expression levels depending on whether they reside in X chromosome or autosomal contexts. Notably, X-autosome translocations do not cause aberrant suppression of translocated autosomal genes or allow general escape from suppression by translocated X-linked genes. This is best seen in comparisons of the same genes assayed across transgene, transposition, and translocation genotypes: *Act5c* and *CG10920* [53] both escape X suppression in transgenes and transpositions but not in translocations (Tables 1, 2 and 6). From these findings, we infer that escape from X suppression results from separating X chromosome genes from their native sex chromosome-specific context. When autosomal and X-linked genes move as part of large chromosome arm-scale reciprocal translocations, they are not necessarily separated from their larger native chromosomal contexts. We speculate that sex chromosome-specific context is in this case determined by chromatin status in the male germline and/or to residence in the distinct sex chromosome territory or subcompartment of the nucleus. These alternatives are not of course mutually exclusive, as chromatin state and transcriptional activity are often mediated by subnuclear localization [60–62].

In somatic cells, 59% of our testis-specific genes (compared to just 2% of our housekeeping genes) reside in BLACK chromatin, which is characterized by a transcriptionally repressive state and a frequent association with the nuclear lamin B protein (among others; [63]; see also [64]). One possibility is that during spermatogenesis, the testis-specific genes on the X chromosome dissociate less readily from the more transcriptionally quiescent nuclear periphery than those on the autosomes. Whatever the mechanism, a characteristic ~3- to 4-fold transcriptional suppression of the X chromosome is detectable very early in the male germline (in cells enriched for premeiotic spermatogonia) and stably maintained through later stages of spermatogenesis [40]. There is no evidence for a dynamic, primary spermatocyte-specific, sex chromosome-wide down-regulation of gene expression, as might be expected for MSCI ([40,47]; but see [48]). Our translocation experiments also fail to reveal the kinds of aberrant expression expected if MSCI is grossly disrupted. MSCI must therefore be so weak as to be undetectable in whole- and sub-testis dissections [40], or it is altogether absent in the *Drosophila* male germline. We therefore conclude that X suppression is distinct from canonical MSCI.

We identified eight sequence motifs enriched in promoter regions of X-linked testis-specific genes (S4 Table). We focused on the AG[tagg]C motif, the most abundant motif with strong overrepresentation among testis versus housekeeping genes and a strong enrichment on the X chromosome versus autosomes. While this motif bears no resemblance to the dosage compensation GA motif [65], the same sequence motif (or a very similar one) was found independently to be enriched near the TSSs of testis-expressed de novo genes that segregate in natural populations of *D*. *melanogaster* [65]. Our statistical and experimental analyses show that the AG[tagg]C promoter element drives 2- to 4-fold higher expression in testis on both the X chromosome and the autosomes. Stronger expression might be achieved via the recruitment of positive regulators of transcription or of proteins that facilitate relocation of testis-specific genes from the nuclear lamina to less peripheral, more transcriptionally active nucleoplasm. Our test of the AG[tagg]C element’s contribution to testis-specificity revealed an interesting X versus autosome difference: in testes, disruption of the AG[tagg]C element reduces *lacZ* expression for both autosomal and X-linked transgenes; in contrast, in the (somatic) male carcass, disruption of the AG[tagg]C element decreases expression for the autosomal transgene but *increases* expression for the X-linked transgene. This qualitative difference suggests that, for testis genes on the X, functional AG[tagg]C elements may contribute to somatic silencing, with disruption of the AG[tagg]C element releasing it from silencing. The fact that this occurs for the X-linked, but not the autosomal site, raises the possibility of an interaction between the AG[tagg]C element and the somatic sex chromosome dosage compensation system.

**Fig 4:**
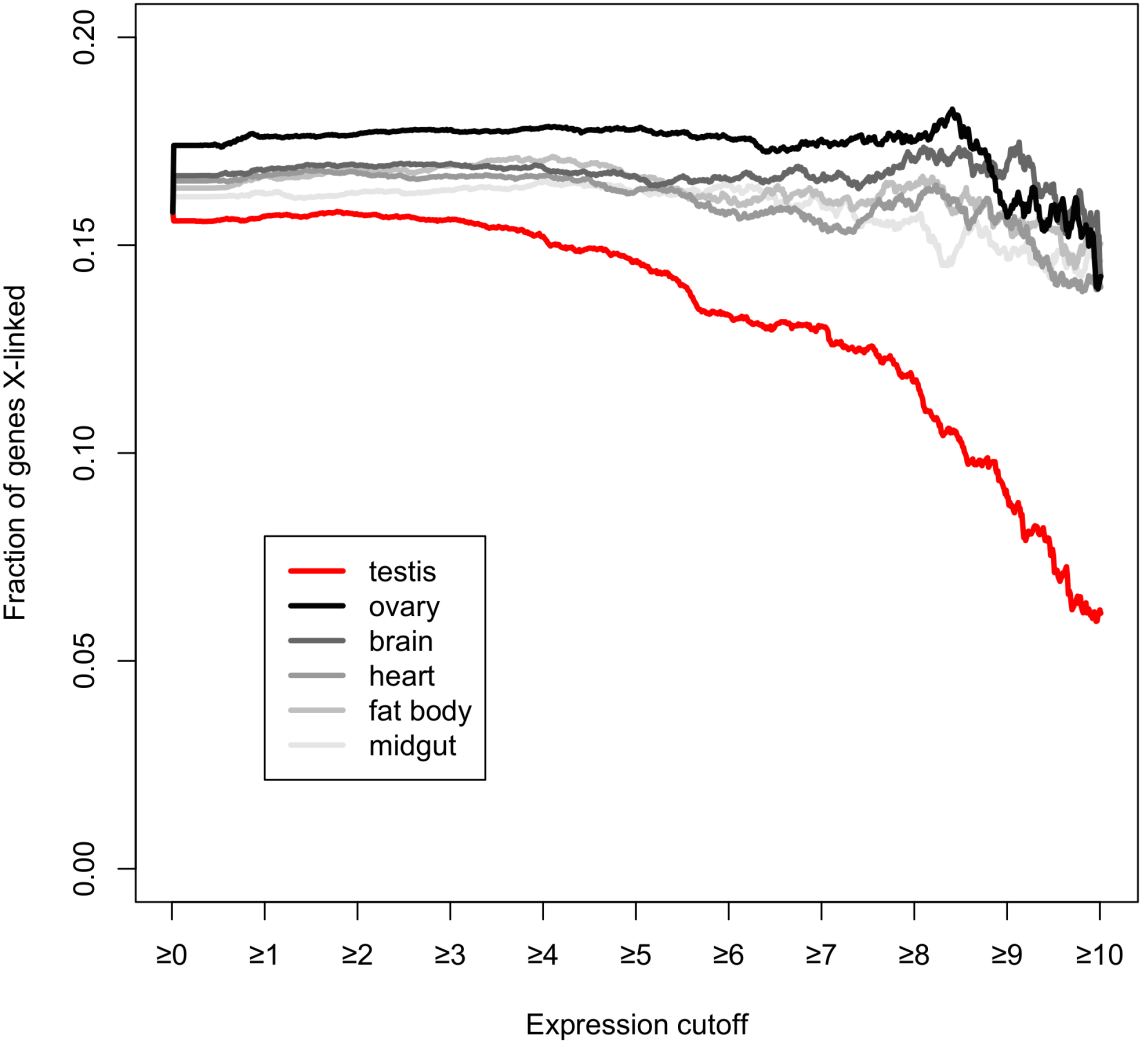
Maximum X-linked gene expression in the testis is constrained. Lines correspond to the fraction of genes with a minimum expression level that are X-linked. For testis (red line), as minimum expression level cut-off increases, the fraction of X-linked genes declines sharply. This decline is not observed for other tissues (gray lines). Gene expression data are from FlyAtlas [69]. The absolute number of genes for each cut-off point is comparable among tissues (not shown). Data found in S1 Data.

The absence of ~3- to 4-fold lower global gene expression from the X chromosome versus the autosomes in wild-type *Drosophila* testes [40,41] indicates that X suppression is compensated, as shown here, by the gene-by-gene recruitment of strong promoters. The balance between chromosome-wide X suppression and compensatory promoters is a curious arrangement, raising the obvious question of why X suppression exists at all—i.e., why would X suppression evolve only for its effects to be cancelled by the evolution of strong promoters? There are at least two broad possibilities. First, X chromosome-wide transcriptional suppression may be an incidental pleiotropic consequence of some other, still unknown phenomenon. Second, X suppression may have evolved deep in the past for reasons that no longer hold and, since then, strong promoters have evolved en masse to compensate. Regardless of its function(s) or its evolutionary history, the constrained transcriptional environment of the X chromosome in the male germline has consequences for gene expression and genome evolution. For instance, X suppression, while generally compensated, may impose an upper limit on the expression level achievable in testis. Consistent with this possibility, we find that the proportion of all X linked genes expressed in testis declines as expression level increases, a pattern that holds equally for testis-specific genes and housekeeping genes (Fig 4; see also [4,66]). X suppression, and the constraint it imposes on maximum expression, may help to explain the genomic distribution of gene duplications. The *Drosophila* genome has an excess of parent genes on the X chromosome that have spawned testis-expressed duplicate genes on the autosomes [67,68]. This pattern of gene duplication may, along with strong promoters, reflect a complementary means to boost expression and compensate for X suppression.

## Materials and Methods

### Fly Strains and Crosses

The *P{wFl-ocn-lacZ}* transgene lines were generously provided by John Parsch (University of Munich). The *T(1;3)OR17* stock was obtained from the Kyoto *Drosophila* Genetic Resource Center, and all other stocks were obtained from Bloomington Stock Center (for the full list, see S4 Table). All flies were raised on standard cornmeal media at 22–23°C.

### Transgene Reporters

We assayed expression of GFP driven by Actin promoters from *P{Act-GFP}* transgenes (n = 5; [70]), and from Ubiquitin promoters from *P{Ubi-GFP}* (n = 4; [71]) and *P{Ubi-GFP(S65T)nls}* transgenes (n = 8; Bloomington stock center). We also assayed expression of mini-white from the *P{wFl-ocn-lacZ}* transgenes (n = 10) [51].

### Transposition Genotypes in Males

We assayed gene expression from two *X/Y; Tp(1;2)/+* transpositions and four synthetic *Df(1)/Y; Dp(1;3)/+* transpositions along with wild-type controls for each. To generate *Tp(1;2)rb*^*+*^*71g* males and *FM6; CyO/+* control males, we crossed *FM6/Y; CyO/Gla* virgin females to *Tp(1;2)rb*^*+*^*71g*, *ct*^6^ *v*^1^ males. *Tp(1;2)rb*^*+*^*71g ct*^1^ *v*^1^*/FM6;CyO* female progeny were then crossed to *y*^1^ w* males. We selected *Tp(1;2)rb*^*+*^*71g*, *ct*^1^, *v*^1^/*Y* and *FM6/Y;CyO/+* males for gene expression assays (see below). Similarly, to generate *Tp(1;2)sn*^*+*^*72d* males and FM6/w*; CyO/+ control males, *FM6;CyO/Gla* virgin females were crossed with *Tp(1;2)sn*^*+*^*72d*, *f*^1^ *car*^1^ males. *Tp(1;2) sn*^*+*^*72d*, *f*^1^ *car*^1^/*FM6;CyO* female progeny were crossed to *y*^1^ w* males. We selected *Tp(1;2)sn*^*+*^*72d*, *f*^1^ *car*^1^*/Y* males and *FM6/Y;CyO/+* males for gene expression assays (see below).

To transpose region 1A1–1A3, we crossed *Df(1)BSC843 w*^1118^*/Binsinscy* females with *w*^1118^*; Dp(1;3)DC004*, *PBac{DC004}VK00033/TM6C*, *Sb*^1^ males. Experimental *Df(1)BSC843*, *w*^1118^*; Dp(1;3)DC004*, *PBac{DC004}VK00033/+* males and control *Binsinscy; TM6C*, *Sb*^1^*/+* males were recovered, and referred to as *Df-Dp(1;3)1A1-1A3* males and control males, respectively. To transpose 4F4-4F5 to 3L, *w*^1118^*; Dp(1;3)DC130*, *PBac{DC130}VK00033* females were crossed to *TM3*, *Ser*^1^*/TM6C*, *Tb*^1^, *Sb*^1^ males, and *w*^1118^*; Dp(1;3)DC130*, *PBac{DC130}VK00033/ TM3*, *Ser*^1^ sons were recovered. These males were crossed to *Df(1)BSC823*, *w*^1118^*/Binsinscy* females. Experimental *Df(1)BSC823*, *w*^1118^*; Dp(1;3)DC130*, *PBac{DC130}VK00033/+* males and control *Binsinscy; TM3*^1^, *Ser*^1^*/+* males were recovered, and referred to as *Df-Dp(1;3)4F4-4F5* males and control males, respectively. To transpose region *6C2-6C8*, *w*^1118^*; Dp(1;3)DC026*, *PBac{DC026}VK00033* females were crossed to *TM3*, *Ser*^1^*/TM6C*, *Tb*^1^, *Sb*^1^ males, and *w*^1118^*; Dp(1;3)DC026*, *PBac{DC026}VK00033/ TM3*, *Ser*^1^ sons were recovered. These males were crossed to *Df(1)BSC535*, *w*^1118^*/FM7h* females. Experimental *Df(1)BSC535*, *w*^1118^*; Dp(1;3)DC026*, *PBac{DC026}VK00033/+* males and control *FM7h; TM3*^*1*^, *Ser*^1^*/+* males were recovered, and referred to as *Df-Dp(1;3)6C2-6C8* and control males, respectively. To transpose region 13F1-13F17, *Df(1)Exel6251*, *w*^1118^ *P{XP-U}Exel6251/FM7c* females were crossed to *w*^1118^*; Dp(1;3)DC523*, *PBac{DC523}VK00033/TM6C*, *Sb*^1^ males. Experimental *Df(1)Excel6251*, *w*^1118^ *P{XP-U}Excel6251; Dp(1;3)DC523*, *PBac{DC523}VK00033/+* males and control *FM7c; TM6C*, *Sb*^1^*/+* males were recovered, and referred to as *Df-Dp(1;3)13F1-13F17* males and control males, respectively. We attempted to generate 17 different autosome-to-X synthetic transpositions as well, but all were inviable.

### Transposition Genotypes in Females

To generate *Tp(1;2)rb*^*+*^*71g* females we crossed FM6/w*; CyO/Gla virgin females to *Tp(1;2)rb*^*+*^*71g*, *ct*^6^ *v*^1^ males. *Tp(1;2)rb*^*+*^*71g ct*^1^ *v*^1^*/FM6;CyO* daughters were crossed to *Tp(1;2)rb*^*+*^*71g*, *ct*^6^ *v*^1^ males, and homozygous *Tp(1;2)rb*^*+*^*71g*, *ct*^6^ *v*^1^ females were selected. To generate *FM6/ y*^1^ w*; CyO/+ control females, we crossed FM6/w*; CyO/Gla virgin females to *Tp(1;2)rb*^*+*^*71g*, *ct*^6^ *v*^1^ males. *Tp(1;2)rb*^*+*^*71g ct*^1^ *v*^1^*/FM6;CyO/* daughters were crossed to *y*^1^ w* males, and *FM6*/ *y*^1^ w*; CyO/+ females were selected.

To generate *Tp(1;2)sn*^*+*^*72d* females we crossed FM6/w*; CyO/Gla virgin females to *Tp(1;2)sn*^*+*^*72d*, *f*^1^ *car*^1^ males. *Tp(1;2)sn*^*+*^*72d*, *f*^1^ *car*^1^*/FM6;CyO* daughters were crossed to *Tp(1;2)sn*^*+*^*72d*, *f*^1^ *car*^*1*^ males, and homozygous *Tp(1;2)sn*^*+*^*72d*, *f*^1^ *car*^1^ females were selected. To generate *FM6/ y*^1^ w*; CyO/+ control females, we crossed FM6/ w*; CyO/Gla virgin females to *Tp(1;2)sn*^*+*^*72d*, *f*^1^ *car*^1^ males. *Tp(1;2)sn*^*+*^*72d*, *f*^1^ *car*^1^ */FM6; CyO* daughters were crossed to *y*^1^ w* males, and *FM6*/ *y*^1^ w*; CyO/+ females were selected.

### Translocations

We attempted to validate the status of seven putative translocation stocks from the *Drosophila* Genetic Resource Center in Kyoto [106092 (*T(1;3)OR60*), 102069 (*T(1;3)OR45)*, 102066 (*T(1;3)sc[260-15T(1;3)OR49)*, 102070 (*T(1;3)OR49*), 102020 (*T(1;2;3)r24)*, 102067 (*T(1;3)OR17*), 102068 (*T(1;3)OR34)*] and six from the Bloomington Stock Center: 3830 (*T(1;3)l-v455)*, 4636 (*T(1;3)GA91)*, 4639 (*T(1;3)JA29)*, 4633 (*T(1;3)GA119)*, 4677 (*T(1;3)GA41)*, 840 (*T(1;3)OR60)*. All translocations except *T(1;3)l-v455* and *T(1;3)OR17* were lost from the stocks prior to arrival in the lab.

### Sample Dissections

All dissections were done in Ringer’s Solution. For all testis samples, seminal vesicles and accessory glands were removed to isolate whole testes. For gonadectomized samples, testes were removed from whole males and ovaries were removed from whole females. All samples were collected from 2–5 day-old mated males or virgin females. For testis dissections, ten testes = one biological replicate; for ovary dissections, two ovaries = one biological replicate; and for carcass dissections, one gonadectomized carcass = one biological replicate. Sheath-removed male germline dissections followed previously published protocols except that here a single dissection included both “apical” and “proximal” material from individual testes [42,50]. Twenty sheath-removed germline dissections = one biological replicate. For the motif transgene experiments, five testes = one biological replicate; and for the corresponding carcass, one gonadectomized male = one biological replicate.

### qRT-PCR Expression Assays

We isolated RNA using the Nucleospin RNA XS kit (Clontech), which includes a DNase step to prevent genomic DNA contamination. cDNA was synthesized from the SuperScript III kit (Invitrogen). All qRT-PCR primers were optimized to 90%–110% efficiency (S7 Table). We determined by Sanger sequencing that the *Actin* and *Ubiquitin* transgenes had different *GFP* alleles. We therefore designed and optimized different qPCR primers for Actin-GFP and Ubiquitin-GFP samples. Whenever possible, primers were designed to span exon-exon junctions to ensure amplification from cDNA. If primers could not be optimized that spanned an exon-exon junction, primers were made that spanned an intron. For all qPCR, a melt curve was performed at the end as a check against spurious amplification. As many testis-specific genes lack introns, the melt curve results of intron-spanning primers from other genes from the same samples provided evidence against genomic DNA contamination. Because control genes are assayed in all experiments, the exon-exon junction-spanning primers in these genes provide controls against genomic DNA contamination in every sample.

For all reactions, 2 μl of cDNA was used in a 20 μl qRT-PCR reaction with SYBR-Green I nucleic acid gel stain (Invitrogen). Two technical replicate qRT-PCR reactions were run for each biological replicate. Ct values were averaged across technical replicate wells for each biological replicate. The mean Ct value for the control genes within each sample was calculated to control for the amount of RNA in each sample. When two control genes were used, the averaged Ct of the mean of the two control genes was used. For synthetic transpositions and Ubi-GFP transgenes, *RpS3* was used as the control gene. For all other samples (except *T(1;3)*) *Rpl32* and *RpS3* were used as control genes. As *Rpl32* and *RpS3* are transposed to the X in *T(1;3)l-v455*, *Rpl24* was used as a control gene for the *T(1;3)* samples. Normalized Ct values for target genes were obtained by subtracting the mean control Ct values from target gene Ct values. For the transposition experiments, five biological replicates were collected for each genotype; for the translocation experiments, three biological replicates were collected for each genotype; and for the motif validation experiments, four biological replicates were collected for each genotype.

### Motif Discovery and Analysis

We used the MEME suite of programs for motif discovery and preliminary analysis. MEME v4.9.0 [58] was used to identify motifs in a focal sequence dataset, while FIMO v.4.9.0 was used to locate occurrences of those motifs in other sequence datasets. MEME was run using the default “zoops” (zero or one occurrence per sequence) model of motif distribution and dirichlet prior on background nucleotide frequencies. We searched for the top ten motifs of size 5–20 bp and allowed motifs to occur on either strand of the sequences in the main discovery dataset. FIMO was run using the search criterion of *p* < 0.0001 for a motif occurrence, allowing for hits to occur on either strand.

The initial discovery sequence dataset used for motif discovery consisted of regions surrounding (-250 bp, +50 bp) transcription start sites of known testis-specific genes on the X chromosome of *D*. *melanogaster*. We restricted our analysis to -250 bp upstream, and +50 bp downstream of the transcription start site, as this region is known to contain core promoter elements [72]. We obtained sequences from the *D*. *melanogaster* genome version r5.51 and expression data from FlyAtlas [69] microarrays as well as RNAseq expression data [73]; FlyBase.org gene-level summaries of tissue-based RNAseq experiments and *D. melanogaster* annotation release 5.50. Testis-specific genes were defined using FlyAtlas data, where genes with specificity measure of *τ* ≥ 0.8 [74] and maximum expression in testes were designated “testis-specific.” For subsequent analyses, we also defined “housekeeping” genes as those broadly expressed across multiple tissues with *τ* ≤ 0.2.

For thoroughness, we used MEME to characterize motif profiles for several different sets of *D*. *melanogaster* genes. These gene sets included: genome-wide housekeeping (*τ* ≤ 0.2) genes; autosomal housekeeping genes; X-linked housekeeping genes; and the same sets (genome-wide, autosomal, X-linked) for testis-specific (*τ* ≥ 0.8) gene sets. The AG[tagg]C motif (or quantitative variants) appeared in all of the motif profiles of testis-specific upstream regions (S2 Fig). For the genome-wide and X-linked gene sets, it appeared as the second most significant motif, while for the autosomal subset it appeared as the fourth most significant motif. No similar motifs appeared in the top ten hits of any of the housekeeping sets, nor did we find the motif enriched in the upstream regions of X-linked genes with highly specific expression (*τ* ≥ 0.8) in tissues other than testis. Finally, we did not recover the AG[tagg]C motif among the top ten hits using a gene set compromising genes highly expressed in, but not specific to, testes (log_2_RPKM ≥ 5, *τ* ≤ 0.8).

### Evolutionary Analysis of the AG[tagg]C Promoter Element

To study the evolution of the AG[tagg]C promoter element, we wrote scripts that extracted -300 bp upstream to +100 bp downstream of TSSs of all genes with at least one motif hit and used BLAST (v.2.2.28+) to identify putatively homologous sequences in *D*. *yakuba* (Flybase, genome version r1.3). Genes with no hits or multiple HSPs were removed from the analysis. For each successful (400 bp) BLAST we reconstructed as much of the smaller 300 bp region used in motif-finding (-250 bp upstream to +50 bp downstream of the TSS) as could be clearly aligned by BLAST between the two species. For each instance of a motif found in the *D*. *melanogaster* sequences, we extracted the corresponding putatively homologous sites in *D*. *yakuba* by sequence coordinates within the 300 bp region. We then calculated divergence at each of the 19 motif positions, counting single-base indels (~9%–10% of all changes) as single events. For each of the 19 motif positions, we used the initial MEME search description of the motif to calculate a “position information” score as 2-Σf_i_log_2_(f_i_), with f_i_ the frequency of the i^th^ nucleotide found at a position. The position information score corresponds to the summed height of the four letters at a position in the MEME logo, and, ranging between 0 (for four equally frequent nucleotides) to 2 (for a single invariant nucleotide) gives a sense of the conservation of the position within the motif occurrences. In addition to the position-specific motif divergence, we also calculated overall divergence at positions inside identified motifs and outside identified motifs.

### Validation of the AG[tagg]C Promoter Element

We validated a promoter motif using site-directed mutagenesis and transgenic assays. First, we PCR-amplified 766 bp upstream of the testis-specific gene, *CG12681*, using forward 5′ CAA ATT ACG TTT CAT TAC GC and reverse 5′ CAA ATT TCC GTA CTT AAT G primers. The amplicon was cloned into TOPO pCR2.1 vector (Life Technologies) and transformed into frozen competent Top10 cells (Invitrogen). We PCR-screened transformed cells, sequenced clones to check for PCR mutations, and then purified plasmid DNA for use in site-directed mutagenesis. We altered nucleotide states at multiple positions in the wild-type sequence (see Table 3), using the following primers: B2 forward 5′ GCG GCC ACT GTG GAA AGT GTA ATC GCT GTC AG; B2 reverse 5′ GAT TAC ACT TTC CAA GTG GCC GCA AGA AAA TG; B5 forward 5′ GCG GCC AAG TGG GAA GTG TAA TCG CTG TCA G; B5 reverse 5′ GAT TAC ACT TCC CAC TTG GCC GCA AGA AAA TG; A5 forward 5′ TGT AAG TTT AAA AGT GGT TGC CCA TCC GTG TG; A5 reverse 5′ GCA ACC ACT TTT AAA CTT ACA TTT TCC GTT GG; AB forward 5′ GAC TTG GTT GAG TAC TCA CCG TCA C; AB reverse 5′ GTG ACT GGT GAG TAC TCA ACC AAG TC.

The PCR amplicons were digested with *DpnI* (NEB) and transformed into *Top10* competent cells by Gibson cloning (Invitrogen). Each plasmid was subsequently opened with *NotI* (NEB) and phosphatased with Fast AP (Thermoscientific), to prevent vector religation. pCMV-sport (Life Technologies) was also digested with *NotI* to obtain the 3.4 kb *lacZ* fragment. *lacZ* was then ligated into each of five plasmids—four with mutant *CG12681* promoters and one with wild-type *CG12681* promoter. The pCMV-sport[*CG12681-lacZ*] plasmids were transformed into chemically competent Top10 cells and verified by restriction digests and sequencing. We next subcloned the *CG12681*-*lacZ* sequences into *P[acman]-Ap*^*r*^ *F-2-5-attB* vectors (hereafter, *attB* [59], donated by Hugo Bellen [Baylor College of Medicine] and distributed to us by the Drosophila Genome Resource Center). We digested each of the five pCMV-sport[*CG12681-lacZ*] plasmids with *SpeI*, *ScaI*, and *XhoI*, (NEB) with the sticky ends filled-in with Klenow; the resulting 4.3 kb fragments were ligated into the *attB* vector previously cut with *SpeI* and phosphatased with Fast AP. Ligations were electroporated into Epi300 frozen competent cells (Epicentre), clones were verified by restriction digest and sequencing, and the new *attB* constructs were isolated using an Endo Free Qiagen Maxi kit. The *attB* constructs were injected into embryos from two stocks, one with an *attP* landing site on the *X* chromosome (genomic coordinate X:5,757,560) and another on *3L* (cytological position 75A10; genomic coordinate 3L:17,952,108) at BestGene (http://www.thebestgene.com). Finally, we confirmed the transformation status and promoter sequences of all transgenic fly lines by a further round of sequencing of the *CG12681* promoter.

## Supporting Information

S1 Data qRT-PCR raw data. (XLS)

S2 Data Computational data for motif analysis. (XLSX)

S1 Fig Wild-type and X-autosome translocation genotypes. (A) Wild-type *D*. *melanogaster* genotype, with X and Y chromosomes shown in blue (Y is smaller, hooked), and chromosome 3 shown in gray. (B) *T(1;3)l-v455* genotype with approximate breakpoints shown. X chromosome cytological positions 1–3C translocated to division 81 on *3R*, and *3R* regions 81–100 translocated to subdivision 3C on the X. (C) *T(1;3)OR17* genotype with approximate breakpoints shown. X chromosome regions 1–19E are translocated to region 67C on *3L*. Chromosome arm *3L* regions 61–67C are translocated to subdivision 19E on the X. Tick marks show the approximate locations of 32 genes whose expression was assayed in the testes by qPCR from wild-type and translocation males is shown. Tick marks above chromosomes indicate genes also assayed in transposition experiments, those below were assayed only in translocation experiments. Red and gray tick marks indicate testis-specific and broadly-expressed genes, respectively. Data found in S1 Data.
(TIF)

S2 Fig Recovery of the AG[tagg]C motif in MEME analyses of upstream (-250,+50) regions depends on the particular genes sets of *D*. *melanogaster* genes. Overall, we searched genome-wide housekeeping (*τ* ≤ 0.2) genes; autosomal housekeeping genes; X-linked housekeeping genes; and the same sets (genome-wide, autosomal, X-linked) for testis-specific (*τ* ≥ 0.8) genes. The AG[tagg]C motif is recovered in all motif profiles of testis-specific upstream regions: for the genome-wide (A) and X-linked sets (B), the AG[tagg]C motif is the second-most significant motif, whereas for the autosomal subset it appears as the fourth-most significant motif (C). No similar motifs appeared in the top ten hits of any of the housekeeping sets. Data found in S2 Data.
(TIF)

S3 Fig AG[tagg]C motif divergence at housekeeping genes. Divergence between *D*. *melanogaster* and *D*. *yakuba* at individual positions of *n* = 62 (coding-oriented) motifs discovered in *D*. *melanogaster* housekeeping (*τ* ≤ 0.2) gene upstream regions. Linear regression of sequence divergence on position information (letter height) was non-significant, *p* = 0.588. See Materials and Methods for details. Data found in S2 Data.
(TIF)

S4 Fig (A) The *Drosophila melanogaster* gene *CG12681* with the intronless CDS (blue), 5′- and 3′-UTRs (gray), and distal and proximal upstream AG[tagg]C motifs (red) at positions -193 bp and -51 bp, respectively, of the transcription start site. Two arrows indicate approximate positions of forward and reverse primers used to generate a 766 bp amplicon from the upstream noncoding region of *CG12681* (see Materials and Methods for details). Wild-type and experimentally altered sequences are shown, with nucleotides changed by site-directed mutagenesis shown in red font. (B) Wild-type or mutant *CG12681* promoters plus *lacZ* reporter sequences were cloned into the *SpeI* multiple cloning site of the *P[acman]-Ap*^*r*^ *F-2-5-attB* vector. Flies with an X-linked (*X*:5,757,560) and autosomal (*3L*:17,952,108) *attP* landing sites were transformed (see Materials and Methods for details).
(TIF)

S1 Table Expression of X-linked genes in wild-type versus transposition male carcass. (XLS)

S2 Table Expression of X-linked genes in wild-type versus transposition female carcass. (XLS)

S3 Table Expression of X-linked genes in wild-type versus transposition ovary. (XLS)

S4 Table Expression of *P{Ubi-GFP*.*D}* in additional tissues. (XLS)

S5 Table Motif enrichment by chromosome and gene type. FIMO hits (*p* < 0.0001) of motifs in testis-specific, tissue-specific (non-testis), and housekeeping genes of the *D*. *melanogaster* genome (annotation release r5.51), split by chromosome type. For each category, results are given in: number of sequences with at least one hit, and (in parentheses) sequences with a hit/number of sequences.
(DOC)

S6 Table Fly stocks used in experiments. (XLS)

S7 Table qRT-PCR primers and efficiencies. (XLS)

## Abbreviations

*Act5c*: *Actin 5c*
MRE: MSL recognition element
MSCI: meiotic sex chromosome inactivation
MSL: Male-Specific Lethal
qRT-PCR: quantitative real time PCR
TSS: transcription start sites
*Ubi*: *Ubiquitin*
