# Supplementary figures and images for: Sex Chromosome-wide Transcriptional Suppression and Compensatory Cis-Regulatory Evolution Mediate Gene Expression in the Drosophila Male Germline
Source: PLoS Biol. 2016 Jul 12;14(7):e1002499. doi: 10.1371/journal.pbio.1002499 (PMC4942098; doi:10.1371/journal.pbio.1002499)

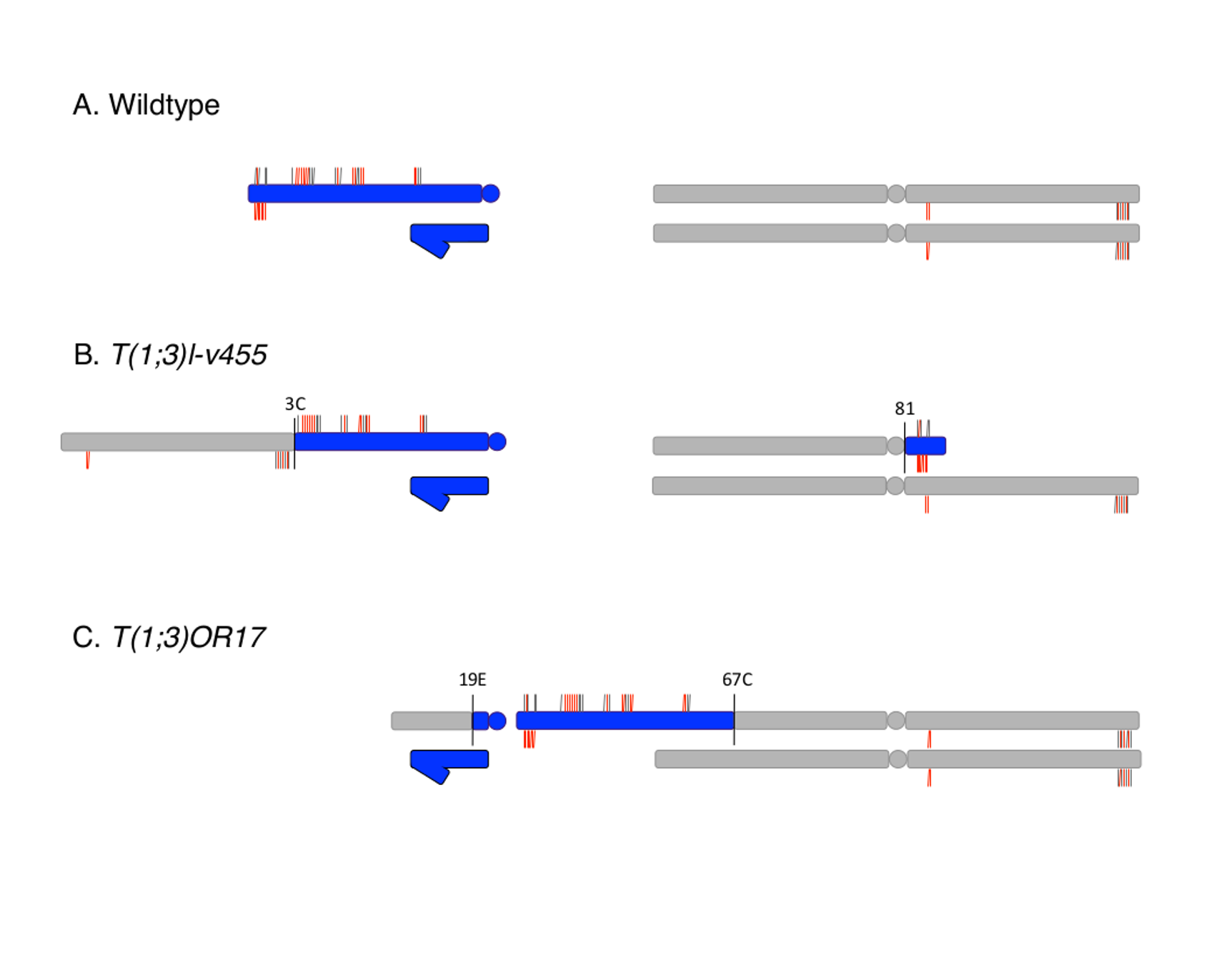

Supplement: S1 Fig — (A) Wild-type D. melanogaster genotype, with X and Y chromosomes shown in blue (Y is smaller, hooked), and chromosome 3 shown in gray. (B) T(1;3)l-v455 genotype with approximate breakpoints shown. X chromosome cytological positions 1–3C translocated to division 81 on 3R, and 3R regions 81–100 translocated to subdivision 3C on the X. (C) T(1;3)OR17 genotype with approximate breakpoints shown. X chromosome regions 1–19E are translocated to region 67C on 3L. Chromosome arm 3L regions 61–67C are translocated to subdivision 19E on the X. Tick marks show the approximate locations of 32 genes whose expression was assayed in the testes by qPCR from wild-type and translocation males is shown. Tick marks above chromosomes indicate genes also assayed in transposition experiments, those below were assayed only in translocation experiments. Red and gray tick marks indicate testis-specific and broadly-expressed genes, respectively. Data found in S1 Data. (TIF) [file pbio.1002499.s003.tif]

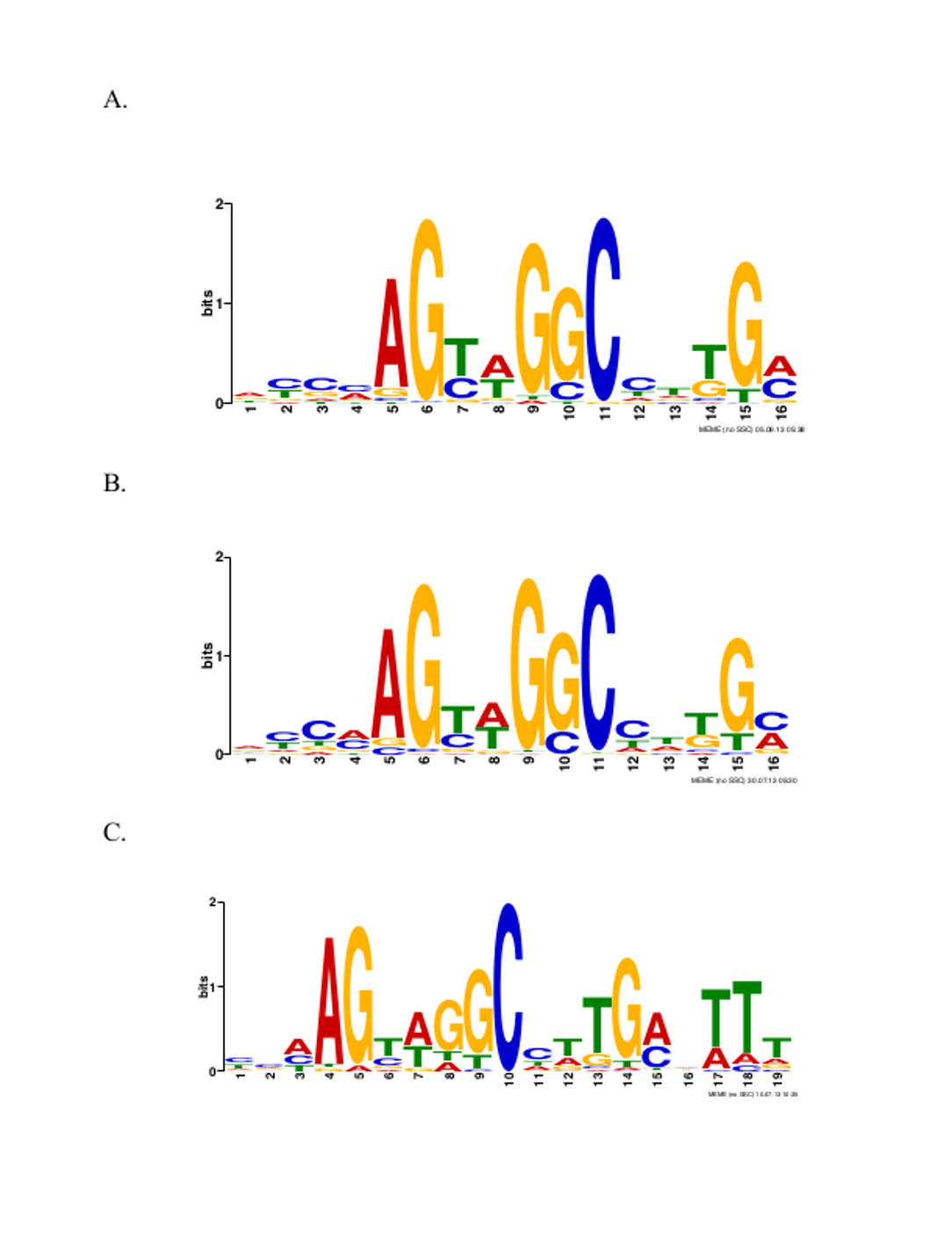

Supplement: S2 Fig — Overall, we searched genome-wide housekeeping (τ ≤ 0.2) genes; autosomal housekeeping genes; X-linked housekeeping genes; and the same sets (genome-wide, autosomal, X-linked) for testis-specific (τ ≥ 0.8) genes. The AG[tagg]C motif is recovered in all motif profiles of testis-specific upstream regions: for the genome-wide (A) and X-linked sets (B), the AG[tagg]C motif is the second-most significant motif, whereas for the autosomal subset it appears as the fourth-most significant motif (C). No similar motifs appeared in the top ten hits of any of the housekeeping sets. Data found in S2 Data. (TIF) [file pbio.1002499.s004.tif]

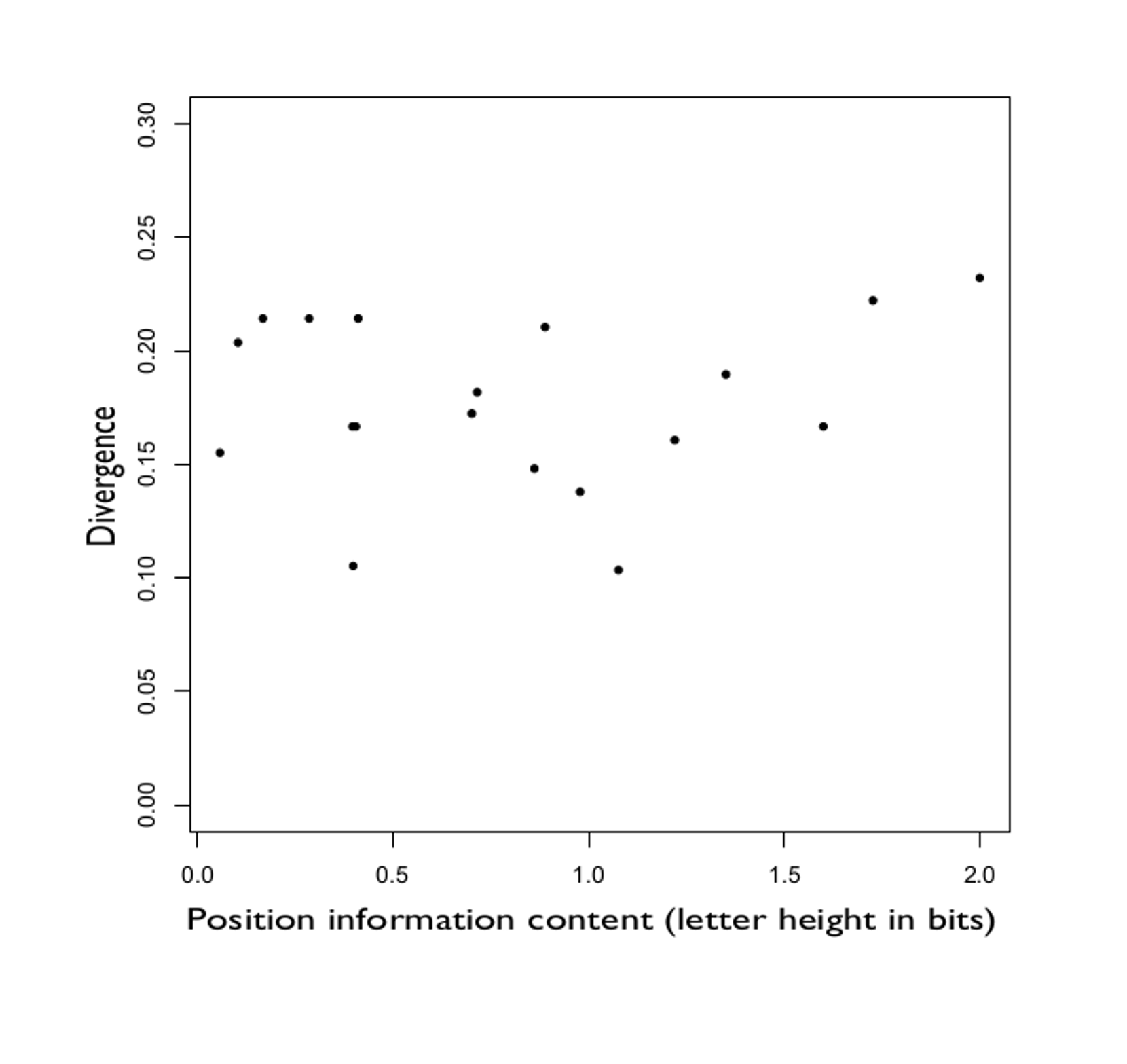

Supplement: S3 Fig — Divergence between D. melanogaster and D. yakuba at individual positions of n = 62 (coding-oriented) motifs discovered in D. melanogaster housekeeping (τ ≤ 0.2) gene upstream regions. Linear regression of sequence divergence on position information (letter height) was non-significant, p = 0.588. See Materials and Methods for details. Data found in S2 Data. (TIF) [file pbio.1002499.s005.tif]

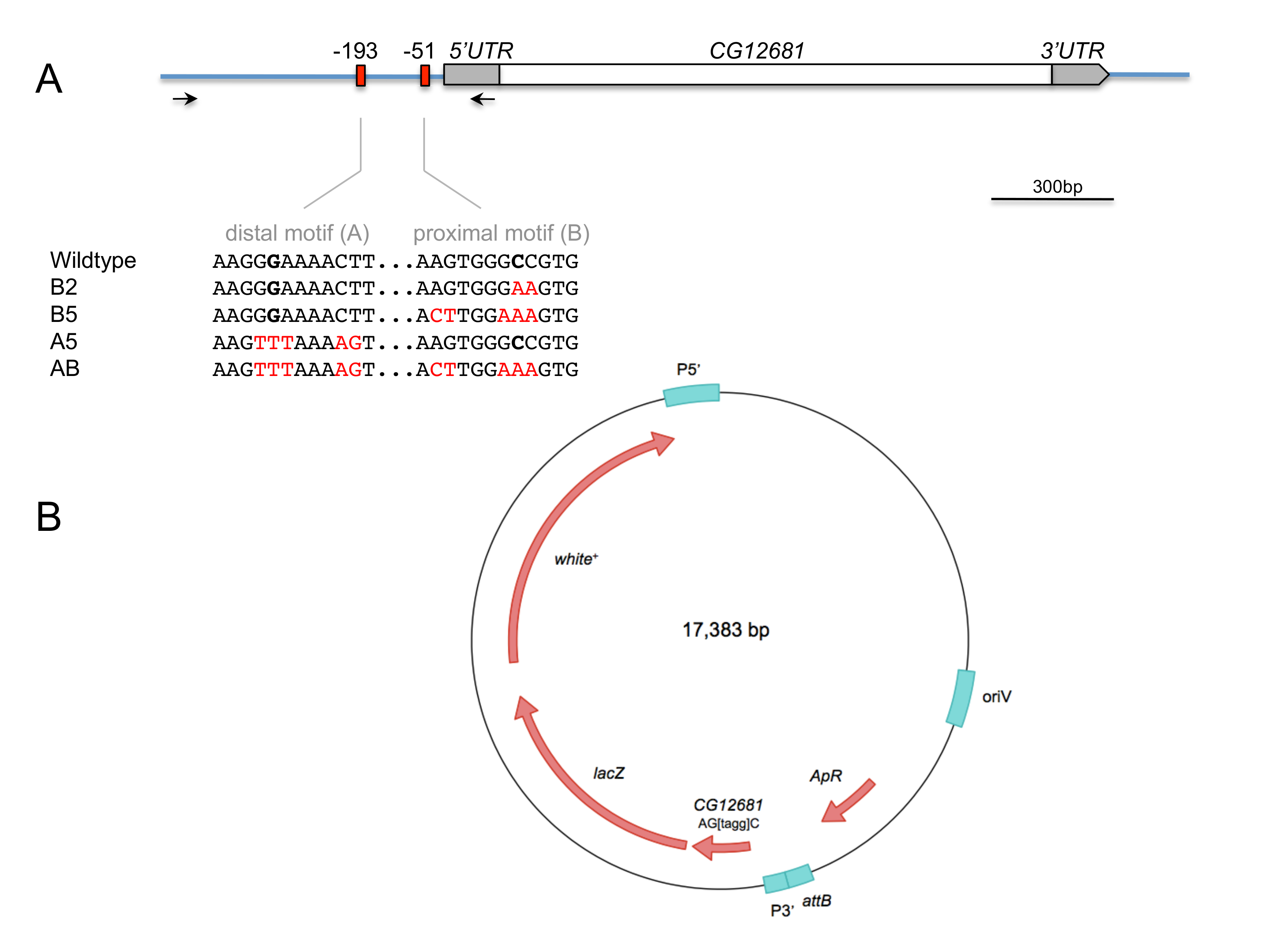

Supplement: S4 Fig — (A) The Drosophila melanogaster gene CG12681 with the intronless CDS (blue), 5′- and 3′-UTRs (gray), and distal and proximal upstream AG[tagg]C motifs (red) at positions -193 bp and -51 bp, respectively, of the transcription start site. Two arrows indicate approximate positions of forward and reverse primers used to generate a 766 bp amplicon from the upstream noncoding region of CG12681 (see Materials and Methods for details). Wild-type and experimentally altered sequences are shown, with nucleotides changed by site-directed mutagenesis shown in red font. (B) Wild-type or mutant CG12681 promoters plus lacZ reporter sequences were cloned into the SpeI multiple cloning site of the P[acman]-Apr F-2-5-attB vector. Flies with an X-linked (X:5,757,560) and autosomal (3L:17,952,108) attP landing sites were transformed (see Materials and Methods for details). (TIF) [file pbio.1002499.s006.tif]
