# Supplementary material for: Sex Chromosome-wide Transcriptional Suppression and Compensatory Cis-Regulatory Evolution Mediate Gene Expression in the Drosophila Male Germline
Source: PLoS Biol. 2016 Jul 12;14(7):e1002499. doi: 10.1371/journal.pbio.1002499 (PMC4942098; doi:10.1371/journal.pbio.1002499)
Supplement: S5 Table — FIMO hits (p < 0.0001) of motifs in testis-specific, tissue-specific (non-testis), and housekeeping genes of the D. melanogaster genome (annotation release r5.51), split by chromosome type. For each category, results are given in: number of sequences with at least one hit, and (in parentheses) sequences with a hit/number of sequences. (DOC) [file pbio.1002499.s011.doc]

|  | Testis-specific (*τ* ≥0.8) | | Tissue-specific (non-testis) (*τ* ≥ 0.8) | | Housekeeping (*τ* ≤ 0.2) | |
| --- | --- | --- | --- | --- | --- | --- |
|  | X  (n=241) | Autosome  (n=1324) | X  (n=361) | Autosome  (n=2208) | X  (n=399) | Autosome  (n=2000) |
| 1. 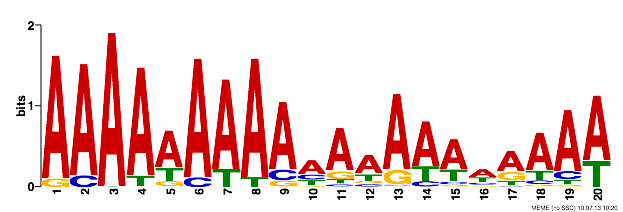 | 138  (0.57) | 645  (0.49) | 133  (0.37) | 697  (0.32) | 238  (0.60) | 1142  (0.57) |
| 2. 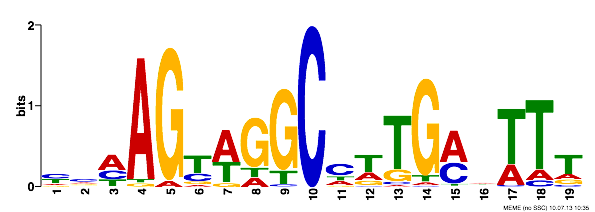 | 78  (0.32) | 253  (0.19) | 26  (0.07) | 145  (0.07) | 19  (0.05) | 152  (0.08) |
| 3. 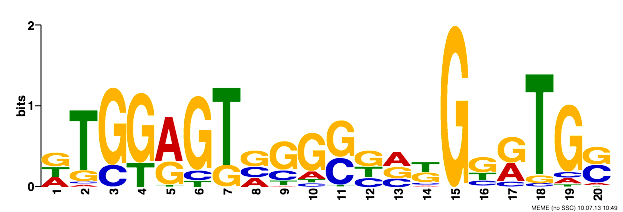 | 30  (0.12) | 39  (0.03) | 29  (0.08) | 154  (0.07) | 15  (0.04) | 64  (0.03) |
| 4. 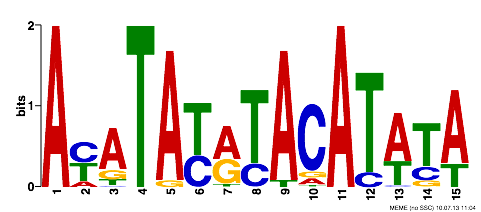 | 79  (0.33) | 287  (0.22) | 67  (0.19) | 328  (0.15) | 98  (0.25) | 461  (0.23) |
| 5. 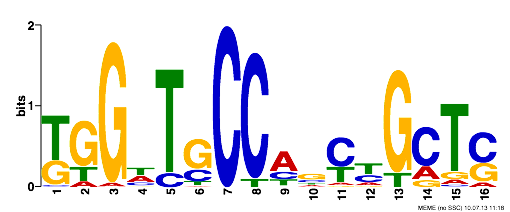 | 34  (0.14) | 69  (0.05) | 29  (0.08) | 165  (0.07) | 31  (0.08) | 121  (0.06) |
| 6. 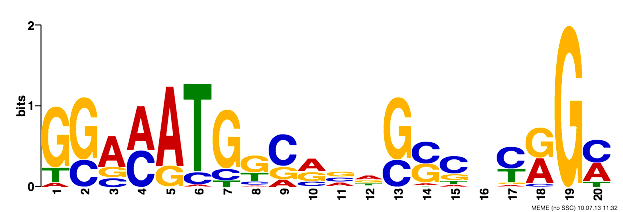 | 40  (0.17) | 68  (0.05) | 41  (0.11) | 224  (0.10) | 32  (0.08) | 98  (0.05) |
| 7. 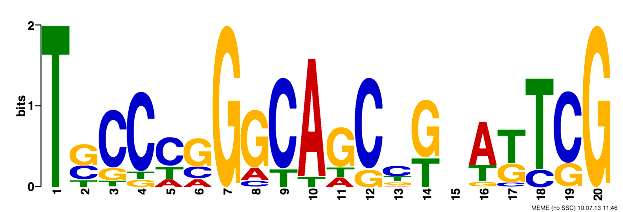 | 19  (0.08) | 64  (0.05) | 36  (0.10) | 165  (0.07) | 16  (0.05) | 85  (0.04) |
| 8. 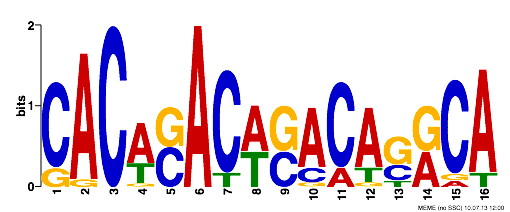 | 31  (0.13) | 105  (0.08) | 52  (0.14) | 204  (0.09) | 32  (0.11) | 155  (0.08) |
